# Ginseng-derived nanoparticles alleviate inflammatory bowel disease via the TLR4/MAPK and p62/Nrf2/Keap1 pathways

**DOI:** 10.1186/s12951-024-02313-x

**Published:** 2024-02-01

**Authors:** Song Yang, Wenjing Li, Xueyuan Bai, Giada Di Nunzio, Liangliang Fan, Yueming Zhao, Limei Ren, Ronghua Zhao, Shuai Bian, Meichen Liu, Yuchi Wei, Daqing Zhao, Jiawen Wang

**Affiliations:** 1 Changchun University of Chinese Medicine, 1035 Boshuo Road, Changchun, 130117 Jilin China; 2 Division of Cardiovascular Medicine, Department of Medicine, Solna, Karolinska Institutet, 17176 Stockholm, Sweden

**Keywords:** Ginseng-derived nanoparticles, Intestinal inflammation, Intestinal flora, Oxidative stress

## Abstract

Inflammatory bowel disease (IBD) is closely linked to the homeostasis of the intestinal environment, and exosomes can be used to treat IBD due to their high biocompatibility and ability to be effectively absorbed by the intestinal tract. However, Ginseng-derived nanoparticles (GDNPs) have not been studied in this context and their mechanism of action remains unclear. Here, we investigated GDNPs ability to mediate intercellular communication in a complex inflammatory microenvironment in order to treat IBD. We found that GDNPs scavenge reactive oxygen species from immune cells and intestinal epithelial cells, inhibit the expression of pro-inflammatory factors, promote the proliferation and differentiation of intestinal stem cells, as well as enhancing the diversity of the intestinal flora. GDNPs significantly stabilise the intestinal barrier thereby promoting tissue repair. Overall, we proved that GDNPs can ameliorate inflammation and oxidative stress in vivo and in vitro, acting on the TLR4/MAPK and p62/Keap1/Nrf2 pathways, and exerting an anti-inflammatory and antioxidant effect. GDNPs mitigated IBD in mice by reducing inflammatory factors and improving the intestinal environment. This study offers new evidence of the potential therapeutic effects of GDNPs in the context of IBD, providing the conceptual ground for an alternative therapeutic strategy.

**Graphical Abstract:**

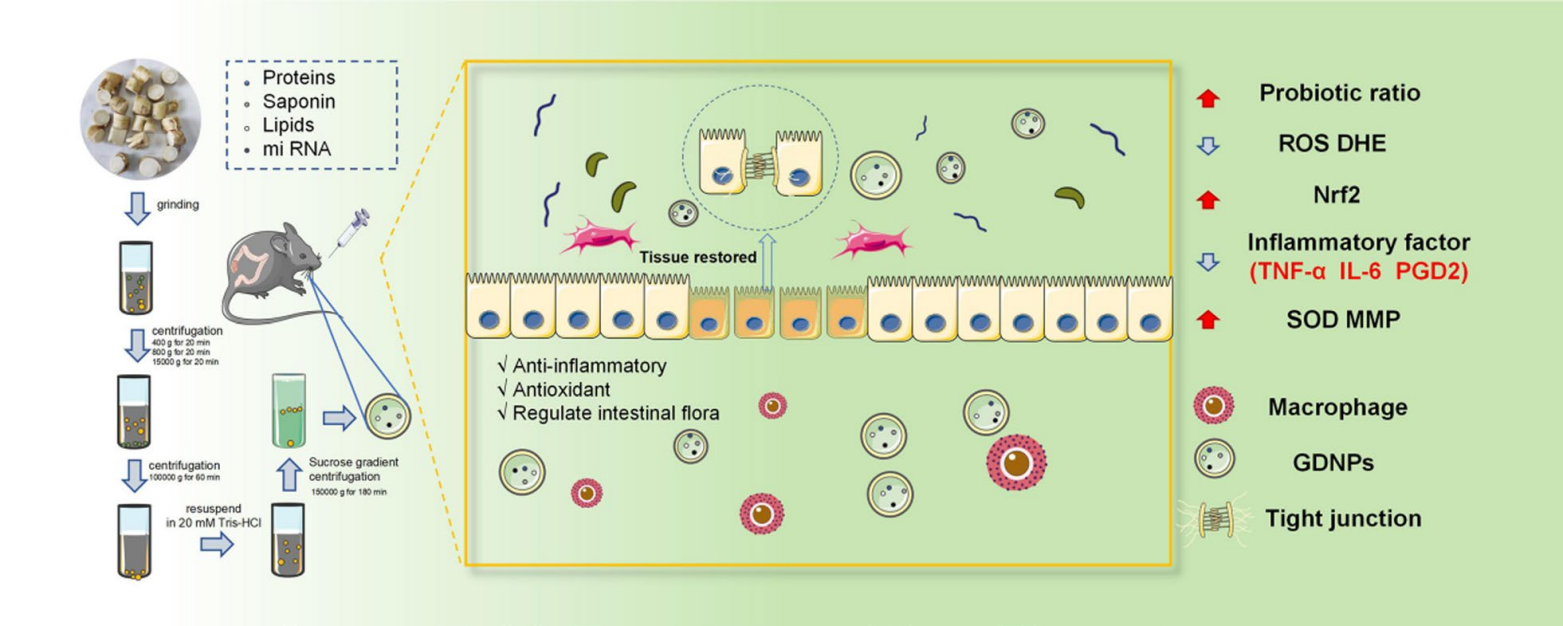

**Supplementary Information:**

The online version contains supplementary material available at 10.1186/s12951-024-02313-x.

## Highlights

- New ideas and high compatibility of plant nanoparticle materials used in clinical medicine.
- Critical components and detailed mechanisms were identified through multi-omics analyses.
- Activation of the body's first line of defence: immune cells—anti-inflammatory pathway—TLR4/MAPK, antioxidant pathway—p62/Nrf2/Keap1 to defend against the complex inflammatory environment.
- Promotion of the proliferation and differentiation of intestinal stem cells by activating the Wnt/β-catenin signaling pathway, reduction of intestinal epithelial inflammation and restoration of intestinal barrier function.
- Regulation of the intestinal flora composition.

**Supplementary Information:**

The online version contains supplementary material available at 10.1186/s12951-024-02313-x.

## Introduction

Inflammatory bowel disease (IBD) includes Crohn’s disease and ulcerative colitis [1]. Millions of individuals are estimated to suffer from IBD worldwide, and the incidence is increasing yearly. IBD has become a global burden due to its severe effects on patient quality of life [2]. Nevertheless, the mechanism behind IBD pathogenesis is still unclear. Intestinal inflammation usually manifests as increased levels of intraepithelial lymphocytes accompanied by the infiltration of various inflammatory immune cell types and surface epithelial damage. The migration of erythrocytes and inflammatory cells into the lamina propria leads to their entrapment in the connective tissue [3]. Increasing evidence points to immune system dysfunction as a central element in the development of IBD [4]. Innate immune cells, especially macrophages, are essential in treating intestinal inflammation. Stem cell therapy of IBD, among other things, relies on regulation of macrophages inflammatory functions. The intestinal mucosal immune system balances pro- and anti-inflammatory mediators [5, 6]. Various intestinal diseases, such as inflammatory bowel disease, can disrupt the intestinal balance and interfere with the continuous self-renewal of the intestinal epithelium [7]. Several lines of evidence points to oxidative stress, inflammation, environmental factors and dysbiosis of the intestinal flora as key factors in the development of intestinal diseases [8–11]. The present study investigates the mitigating effects of GDNPs on inflammatory bowel disease, specifically focusing on the role of macrophages, intestinal epithelial cells, intestinal stem cells and the intestinal microenvironment.

Ginseng (Panax L.) belongs to the family of Wujia and is one of the most renown medicinal herbs worldwide, used for medicinal purposes over many centuries because of its many active ingredients [12]. For example, Ginsenoside Rb1 alleviates colitis by activating the HMG-CoA reductase degradation 1 (HRD1) signaling pathway in the endoplasmic reticulum [13]. Ginsenoside Rf possesses anti-inflammatory properties against intestinal diseases due to its ability to suppress the Mitogen-activated protein kinase (MAPK)/Nuclear factor kappa-B (NF-κB) pathway [14]. Qiseng can reduce intestinal flora dysbiosis and the release of inflammatory factors [15]. The active ingredients in ginseng have also been reported to act as antioxidants and anti-inflammatory agents [16, 17].

Inflammation and oxidative stress play essential roles in the development and progression of various disease [18, 19]. Excessive accumulation of reactive oxygen species (ROS) can undermine the body’s defence system against oxidative stress. However, Superoxide dismutase (SOD) can rescue the damage caused by the accumulation of ROS. Studies have shown that SOD is critical in inhibiting the oxidative inactivation of Nitric oxide (NO). This prevents nitrite formation and endothelial and mitochondrial dysfunction [20, 21]. In addition, the Kelch Like ECH Associated Protein 1 (Keap1)/Nuclear factor erythroid2-related factor 2 (Nrf2) signaling pathway also has an antioxidant role. Unless activated, Nrf2 forms a complex with Keap1 in the cytoplasm. Under these conditions, Nrf2 is ubiquitinated and later degraded. Upon activation, Nrf2 translocates into the nucleus to exert its antioxidant effects [22, 23].

The MAPK signaling pathway is a fundamental inflammatory pathway where the classical MAP kinase (ERK)1/2 has a significant role in promoting inflammation. Recent studies have shown that inhibition of the MAPK pathway ameliorates chronic inflammatory conditions such as encephalitis, pancreatitis and enteritis [24, 25] by, among other things, inhibiting the release of inflammatory cytokines such as Tumor necrosis factor-ɑ (TNF-ɑ), Interleukin 6 (IL-6), and Interleukin 1β (IL-1β) [26]. Based on these evidences, it is clear that modulating macrophages' role in inflammation and oxidative stress might be an important strategy for the treatment and understanding of chronic inflammatory diseases.

Exosomes are small vesicles of 30–100 nm in diameter that can carry proteins, lipids, and microRNAs [27]. Exosomes mediate intercellular communication by acting on several cell receptors. Plant exosomes have become an important research focus in recent years. Plant exosomes have a similar structure and function to mammalian exosomes [28]. It has been reported that the human gastrointestinal tract can directly absorb edible plant exosome nanoparticles, establishing an efficient way of communication with the external environment [29]. For example, cauliflower nanoparticles effectively ameliorate colitis by activating immune cells [30]. Nanoparticles isolated from grapes and grapefruit can modulate mouse stem cells and macrophages, thus protecting mice from dextran sulfate sodium salt (DSS)-induced inflammatory effects [31]. However, the mechanism by which GDNPs might act in this regard is yet to be elucidated. Therefore, we investigated the impact of GDNPs on macrophages and intestinal epithelial cells and their effect on inflammation in an in vitro inflammatory model. Moreover, the effects of GDNPs on colorectal and intestinal stem cells were explored in a mouse model of IBD. We found that GDNPs positively ameliorate the negative effects caused by inflammation and oxidation. We therefore speculate that GDNPs have the potential to become an essential tool in treating intestinal diseases in the near future.

## Materials and methods

### Isolation and purification of GDNPs

Fresh ginseng was cleaned to remove muddy impurities, cut into small pieces, and homogenised at low temperature with appropriate amount of PBS. The obtained filtrate was supplemented with protease inhibitors and the solution pH was adjusted to 7 with 1 M Tris–HCl. Afterwards, the ginseng crude extract underwent three consecutive centrifugation steps (400*g* for 20 min, 800*g* for 20 min and 15,000*g* for 20 min). The supernatant collected after each step was pulled together and ultracentrifuged at 100,000 g for 1 h. The final precipitate was then resuspended in 20 mM Tris–HCl and subjected to sucrose density gradient centrifugation (1 M sucrose and 2 M sucrose solution) at 150,000*g* for 3 h at 4 °C. The intermediate bands were collected and then washed to finally obtain purified GDNPs, which were assayed for protein concentration.

### Characterisation of GDNPs

Transmission electron microscopy and Zetasizer Nano ZS were used to visualise GDNPs [32]. In vitro*,* digestion of GDNPs was performed using simulated gastric and intestinal digestive juices. In brief, 1 mL of GDNPs in 20 mM Tris–HCl solution was incubated at 37 °C for 60 min after the addition of 1.34 μL of 18.5% w/v HCl and 24 μL of a pepsin solution (80 mg/mL in 0.1 M of HCl) to form a gastric juice. Then, 80 μL of a mixture containing 1.92 mg of bile extract and 0.32 mg of pancreatin (dissolved in 0.1 M NaHCO_3_) was added to the gastric juice. The pH of the solution was adjusted to 6.5 with 1 M NaHCO_3_, which was the intestinal juice. GDNPs were incubated for an additional 60 min in the intestinal juice. Subsequently, the resulting product particle size and surface charge were measured to evaluate the stability of GDNPs.

### Component analysis of GDNPs

A combined multi-omics approach was used to detect components in GDNPs, including lipids, and proteins.

#### Lipid analysis

Lipids were extracted according to the Methyl tert-butyl ether (MTBE) method. Briefly, samples were spiked with an appropriate amount of internal lipid standards and then homogenised with water/methanol (1:1.2, v/v). After the addition of MTBE (0.8 mL per sample) the mixture was extracted by ultrasound. After incubation at room temperature for 30 min, the solution was centrifuged at 14,000×*g* for 15 min at 10 °C. The organic solvent layer (upper layer) was collected and dried under nitrogen stream. Lipid analysis was performed using Liquid chromatography-tandem mass spectrometry (LC–MS/MS) method [33].

#### Lipidomic and proteomic analysis of GDNPs

The obtained GDNPs were sent to the New Life Institute (Shanghai, China) for lipidomic assays. Briefly, the lipids were extracted, separated by ultra-high performance liquid chromatography, and analysed by mass spectrometry. LipidSearch was used for data processing and analysis. The isotopic internal standard method was used to calculate the content of GDNPs using the ratio of the response abundance of GDNPs to the internal standard (peak area ratio) and the concentration of the internal standard. For proteomics analyses, proteins were extracted and subjected to enzymatic digestion, followed by analysis and identification by LC–MS/MS. Mascot software was used for library identification and quantitative analysis. Subcellular localisation analysis of all identified proteins using the subcellular structure prediction software CELLO (http://cello.life.nctu.edu.tw/). The structure domain prediction software “interproscan” was used to predict the structure domains of all identified proteins.

### Ethics and animals

The experimental animals used in this study were C57BL/6J male mice (18–25 g, 8 weeks old), purchased from Changchun Yis and kept at the appropriate temperature and humidity with free access to water and diet. ARRIVE guidelines handled all animal experiments, and all followed the five freedoms. All experimental procedures hereby described have been approved by Ethical Review of Animal Experiments at Changchun University of Traditional Chinese Medicine (Approval No. 2023546).

### In vitro and in vivo GDNPs uptake assays

In vitro uptake assay: RAW264.7 cells were seeded in 6-well plates at 2 × 10^5^ cells/well. The cells were cocultured with 3,3′-dioctadecyloxacarbocyanine perchlorate (DIO) fluorescent dye labelled with 10 µg/mL (in protein concentration) GDNPs for 24 h. Cells were washed three times with phosphate buffer saline (PBS) after staining the nuclei with 2-(4-Amidinophenyl)-6-indolecarbamidine dihydrochloride (DAPI) for 10 min. Cell uptake was observed under a laser confocal microscope.

In vivo uptake assay: Mice were gavaged with PKH26 dye-labelled GDNPs, and the intestines were removed following euthanasia 6 h later. Subsequently; fixation, embedding, staining, and imaging of the intestinal tissue were performed. Colocalization of macrophages (stained for the macrophage marker EMR1 rabbit pAb) with GDNPs was visualised with a fluorescence microscope (Thermo Fisher Scientific, Inc.).

### In vivo distribution and stability analysis

Male C57BL/6J mice were given PKH26-labeled GDNPs by gavage. Mice were sacrificed 6, 12 and 24 h after gavage of GDNPs, and organs were collected for each timepoint. The fluorescence distribution in each organ was detected using the in vivo imaging system (IVIS) series in vivo imaging system (Spectrum, Germany).

### Establishment of inflammation models in vitro

Our in vitro inflammation models were created by treating cells with lipopolysaccharide (LPS).

RAW264.7 cells were cultured in DMEM medium containing 10% FBS and incubated at 95% air/5% CO_2_ at 37 °C. RAW264.7 cells (2 × 10^5^ cells/well) were then cultured overnight in 6-well plates and treated with 1 µg/mL LPS or different concentrations of GDNPs/LPS for 24 h.

Caco-2 cells were maintained in a Caco-2 cell-specific medium and incubated at 37 °C in a humidified atmosphere of 5% CO_2_ and 95% air. 96-well plates with a seeding density of 2 × 10^4^ cells were used to maintain subcultures of Caco-2 cells that were allowed to attach to the plate for 24 h. The cells were then treated with 5 µg/mL LPS only or different concentrations of GDNPs/LPS for additional 24 h.

### Detection of NO and fluorescent staining

The intracellular NO content was detected using a NO kit (Beyotime). Fluorescent probes for Dihydroethidium (DHE, 10 µM), mitochondrial membrane potential [a probe of mitochondrial membrane potential (JC-1), 1x], ROS [2ʹ,7ʹ-Dichlorodihydrofluorescein diacetate (DCFH-DA), 10 µM], and a probe to enable mitochondria visualization (Mitotracker Red CMXRos, 200 nM) were used to detect the corresponding intracellular indicator. All probes were incubated for 20 min at 37 °C. RAW264.7 cells were washed with PBS buffer twice and visualised under a fluorescence microscope. Image processing was performed using ImageJ analysis software. Analyses were performed using GraphPad Prism 6.0 software.

### qRT-PCR

TRIzol (Beyotime) was used to extract total Ribonucleic acid (RNA). Reverse transcription was performed using the TransGen Biotech kit. Quantitative real-time polymerase chain reaction (qRT-PCR) was performed using the SYBR Premix Ex Taq Kit and the CFX Connect™ Real-Time System (Bio-Rad Laboratories, Inc.). The relevant mRNA primers for the target genes are shown in Tables 1, 2, and 3. Data relative to GAPDH expression levels were calculated using the 2^−∆∆Ct^ method.

**Table 1:** Primers used for the qRT-PCR study (RAW264.7)

Gene	Primer (5ʹ to 3ʹ)
IL-6	F:CGGAGAGGAGACTTCACAGAG
	R:CATTTCCACGATTTCCCAGA
GAPDH	F:AAGGTCATCCCAGAGCTGAA
	R:CTGCTTCACCACCTCTTGA
TNF-α	F:TTGTCTACTCCCAGGTTCTCT
	R:GAGGTTGACTTTCTCCTGGTATG
IL-10	F: TGCACTACCAAAGCCACAAG
	R: TCAGTAAGAGCAGGCAGCAT
PGD_2_	F:GGAGGCCAACTATGACGAGT
	R:TCAGAGTCTGGGTTCTGCTG
PGE2	F:GGAGGCCAACTATGACGAGT
	R:GGAGGCCAACTATGACGAGT

**Table 2:** Primers used for the qRT-PCR study (Caco-2)

Gene	Primer (5ʹ to 3ʹ)
GAPDH	F:CACCAACTGCTTAGCACCCC
	R:TGGTCATGAGTCCTTCCACG
TNF-α	F:CCCAGGGACCTCTCTCTAATC
	R:ATGGGCTACAGGCTTGTCACT
IL-1β	F:TGCTCAAGTGTCTGAAGCAG
	R:TGGTGGTCGGAGATTCGTAG
Claudin-1	FTGGTCAGGCTCTCTTCACTG
	R:TTGGATAGGGCCTTGGTGTT
Occludin	F:AAGGGAAGAGCAGGAAGGTC
	R:TCCAGCTCATCACAGGACTC
ZO-1	F:TTCACGCAGTTACGAGCAAG
	R:TTGGTGTTTGAAGGCAGAGC

**Table 3:** Primers used for the qRT-PCR study (Intestinal stem cell)

Gene	Primer (5ʹ to 3ʹ)
GAPDH	F:AAGGTCATCCCAGAGCTGAA
	R:CTGCTTCACCACCTTCTTGA
Bmi-1	F:CCTTTGCCAGTAGACC
	R:AAGTTGCTGATGACCC
CDX1	F:ACGCCCTACGAATGGATG
	R:CTTGCGCCGGATAGTGAT
MUC2	F:CTGGACTTTGGGAATAG
	R:CTGGGTTGTGGCTTAC

### Western blotting

RAW264.7 cells and mouse intestinal tissues were lysed with RIPA lysis solution at 4 °C for 30 min. The lysates were then centrifuged at 12,000×*g* for 10 min to obtain protein samples. Protein concentration was measured, and 5 × loading buffer was added in varying amounts to adjust for protein concentration. The samples were boiled for 10 min. Equal protein amounts (40 µg) were separated by sodium dodecyl sulfate polyacrylamide gel electrophoresis (SDS-PAGE) (70 v, 30 min; 140 v, 50 min). Then, the separated proteins were transferred to nitrocellulose membranes (Pall, Part Washington, NY, USA). After blocking the membrane with 5% skim milk powder dissolved in PBS for 1 h, the corresponding primary antibody was added, and the membrane was incubated overnight at 4 °C with a 1:1000 dilution. Primary antibodies were removed, the membrane was washed three times with phosphate buffered saline with tween-20 (PBST), and then horseradish peroxidase-conjugated IgG anti-rabbit (or mouse) antibodies (Jackson ImmunoReseach, West Grove, PA, USA) were added and incubated at 25 °C for 1 h. After washing with PBST, protein bands were visualised using an ultra-high sensitivity enhanced chemiluminescence (ECL) substrate kit (Beyotime). β-actin was used as an internal reference protein (Bioss Technology, Beijing, China). Densitometric analysis was performed with ImageJ Software (v1.8.0).

### Induction and treatment of IBD

Our in vivo IBD model was established by administering 2.5% DSS solution into the drinking water of C57BL/6J male mice for 7 days. The experimental mice were randomly divided into five groups: blank control group, IBD model group (2.5% DSS solution), positive control group (2.5% DSS solution + 0.3 g/kg of sulfasalazine), low-dose group (2.5% DSS solution + 5 mg/mL of GDNPs), and high-dose group (2.5% DSS solution + 10 mg/mL in protein of GDNPs). After 3 days of pharmacological pre-protection with GDNPs, normal water was replaced with 2.5% DSS solution. Mice consumed DSS ad libitum, while GDNPs were gavaged in a volume of 0.2 mL per mouse. After 7 days, all mice were sacrificed following anaesthesia, and serum and tissues were collected and stored at − 80 °C.

For the evaluation of GDNPs toxicity levels, a control group and a drug treatment group were gavaged for 10 consecutive days with GDNPs (10 mg/mL, 0.2 mL per mouse). Heart, liver, spleen, lung, kidney and different sections of the intestine (stomach, duodenum, jejunum, ileum, cecum, and colon) were harvested for histological analysis. Mouse blood was tested for [TNF-α, IL-6, SOD, Malondialdehyde (MDA)] using ELISA and Beyotime kits. Haematology analysis was performed using a haematology analyser.

### Intestinal crypts isolation

After sacrificing the mice, the colorectum was placed into a Petri dish containing pre-cooled dulbecco’s phosphate-buffered saline (DPBS). Using a 10 mL syringe, the intestine was rinsed to remove the contents. The cleaned intestines were opened longitudinally, the remaining intestinal contents and villi were gently scraped off with coverslips, and the tissue was washed with DPBS. The tissue was then cut into 10 mm fragments that were again washed with DPBS. Once the intestinal fragments settled by gravity to the bottom of the tube, the supernatant in the solution was aspirated with a pipette and the washing step was repeated until the supernatant became a clear solution. The intestinal fragments were then transferred to a tube containing 20 mL of crypt isolation buffer (Tables 4, 5). After incubating the solution at 4 °C for 30 min, the supernatant was aspirated and discarded, while the tissue pellet was resuspended in 10 mL of DPBS containing 10% fetal bovine serum and shaken vigorously until the crypts were released. Using a pipette, the supernatant was gently aspirated and filtered with a 70-mesh filter. The proteins were extracted by adding a RIPA Lysis Buffer (Beyotime).

**Table 4:** Colonic crypt buffer

Colonic crypt isolation buffer	
5 * Chelating stock buffer	4 mL
Distilled water	16 mL
Dithiothreitol [100 mM]	100 μL [0.5 mM]
EDTA [0.5 M]	200 μL [5 mM]

**Table 5:** Chelating stock buffer

	500 mL of distilled water
Na_2_HPO_4_	1.97 g [28 mM]
KH_2_PO_4_	2.7 g [40 mM]
NaCl	14 g [480 mM]
KCl	0.3 g [8 mM]
Sucrose	37.5 g [220 mM]
Sorbitol	25 g [274 mM]

### Intestinal flora

An evaluation of the effect of GDNPs on intestinal microbiota was conducted by collecting faeces from the cecum of mice before sacrifice. Afterwards, the faeces were analysed for 16S rRNA, which was selected to build a community library by sequencing. The broadly conserved primers, 338F (5ʹ-ACTCCTACGGGAGGCAGCA-3ʹ) and 806R (5ʹ-GGACTACHVGGGTWTCTAAT-3ʹ), were used to amplify this region. In summary, the whole genome deoxyribonucleic acid (DNA) was first extracted, then the 16S rRNA gene amplicon and the internal transcribed spacer (ITS) amplicon were sequenced, and the sequence data were analysed by using the network cloud platform (Shanghai Personal Biotechnology Co., Ltd).

### Statistical analysis

Results are presented as mean ± SD. Statistical analysis was performed by GraphPad Prism 8.0.2. Statistical comparisons were made by Student’s t-test or Kruskal–Wallis test or one-way analysis of variance (ANOVA) followed by Dunnett’s post-hoc test. p < 0.05 was considered statistically significant.

## Results

### Isolation and identification of ginseng-derived nanoparticles and lipidomics

**Fig. 1:**
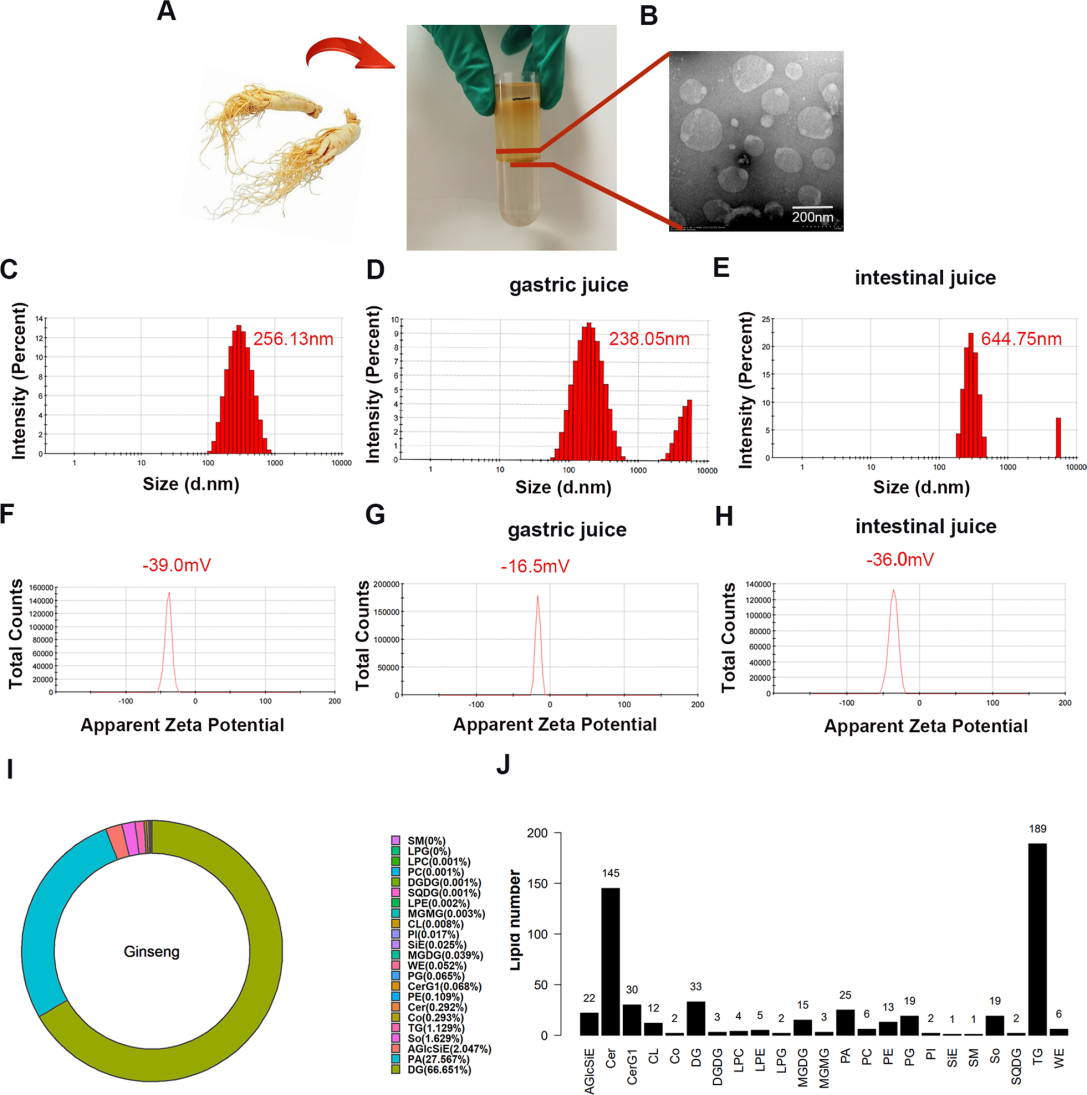
Identification and characterisation of GDNPs. Bands formed after sucrose density gradient ultracentrifugation (**A**). Transmission electron microscopy (TEM) image of GDNPs (**B**). Size distribution (**C**) and surface zeta potential (**F**) of GDNPs particles, measured using a Zetasizer Nano ZS. Changes in size distribution under the action of digestive solutions: gastric juice (**D**) and intestinal juice (**E**). Change in surface zeta potential under the action of gastric juice (**G**) and intestinal juice (**H**). Content expressed in percentages of the different lipid subclasses present in GDNPs particles (**I**). Distribution of lipid class in GDNPs and the number of lipid species in each class (horizontal axis indicates the various lipid class, while the vertical axis indicates the number of lipid species in each subclass) (**J**)

Isolation and purification of GDNPs from ginseng root juice were achieved by differential centrifugation and sucrose density gradient ultracentrifugation (Fig. 1A). The yield of GDNPs (mg of GDNPs protein) per kg of ginseng root was 200–300 mg. Transmission electron microscopy and Zetasizer Nano ZS were used to visualise GDNPs morphology (Fig. 1B) and size. The results indicated that the GDNPs displayed a spherical structure and had an average hydrodynamic particle size of 256 nm (Fig. 1C). The zeta potential of GDNPs was -39.0 mV (Fig. 1F). Furthermore, the stabilities of GDNPs were considered in various digestive fluids. Our results showed that the particle size of GDNPs slightly decreased after digestion with gastric juice (Fig. 1D), and increased after subsequent digestion with intestinal juice (Fig. 1E). After digestion with gastric juice, the surface charge of GDNPs increased from − 39.0 to − 16.5 mV (Fig. 1G), then it decreased to approximately − 36.0 mV (Fig. 1H) after digestion with intestinal juice. This suggests that physiological processes of gastrointestinal digestion affect the particle size and surface potential of GDNPs.

GDNPs contain several lipid subclasses, and are particularly enriched in diacylglycerol (66.67%) and phosphatidic acid (PA) (27.56%) (Fig. 1I). A total of 23 lipid subclasses were detected in GDNPs by lipidomics analysis, for a total number of 559 species. The content and structure of lipid subclasses were determined (Additional file 1: Figure S1). Among them, 189 species were classified as triglycerides, and 145 were classified as ceramides (Fig. 1J). Both are essential intermediates in lipid metabolism and play a crucial role in lipolysis. PA, abundantly present in GDNPs, has been shown to regulate metabolism, signaling, and cell activity in mammalian organisms. PA is closely linked to the MAPK pathway and has a role in stimulating vesicle transport [34, 35].

### Proteomic analysis of GDNPs

**Fig. 2:**
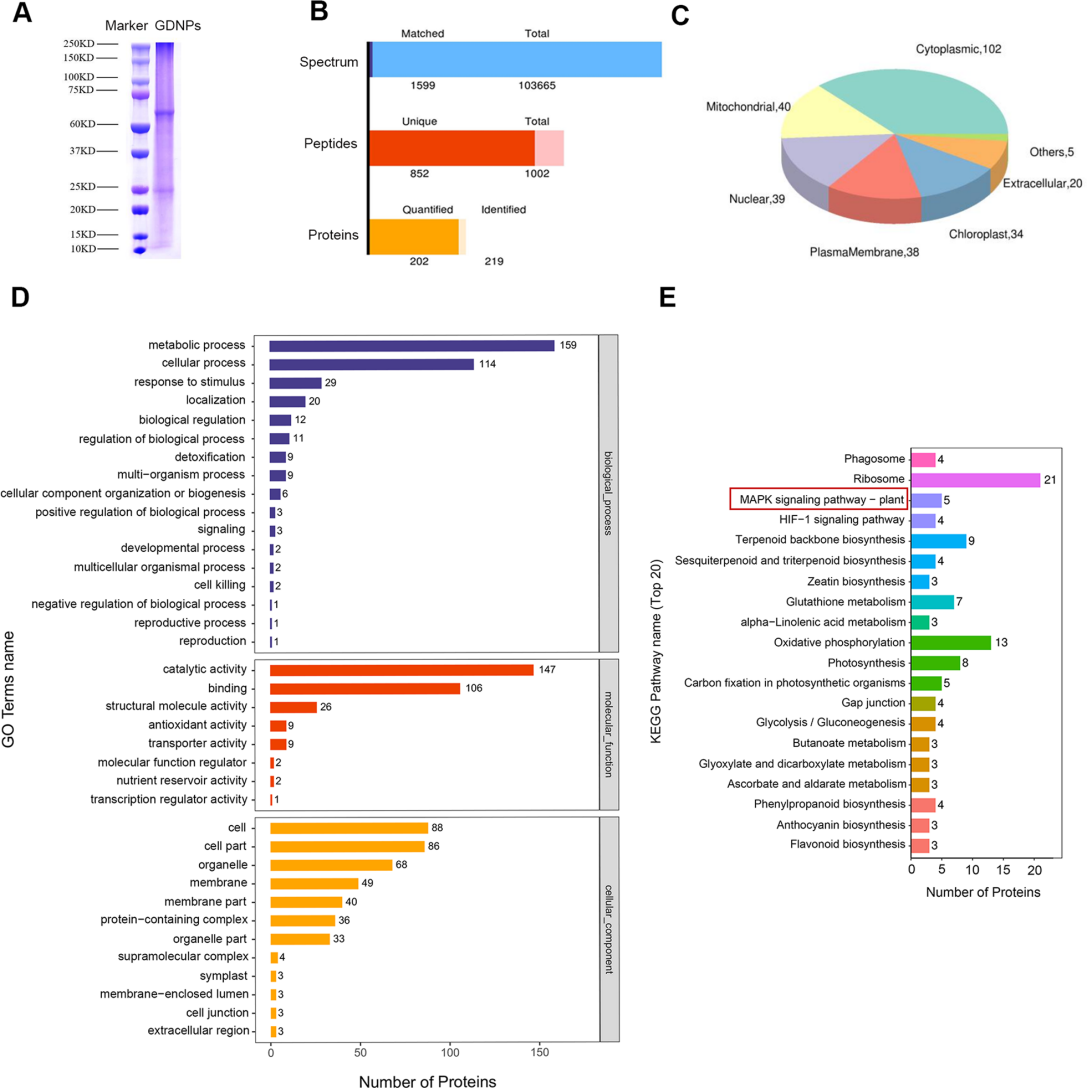
Characterization of GDNPs—Proteomics. Protein gel electrophoresis of GDNPs proteins (**A**). Histogram showing the number of spectra, peptides and proteins quantified and identified in GDNPs (**B**). Pie chart representing the subcellular localisation of the identified GDNPs proteins (**C**). GO annotation analysis (**D**). KEGG pathway annotation analysis (**E**)

Proteomics allows for the characterization of proteins on a large scale, including their expression levels and interactions. Proteomics is indicative of disease development, cellular metabolism, and other processes. The proteins within GDNPs had a molecular weight between 60 and 75 kDa (Fig. 2A). Analysis of GDNPs showed numerous spectra, and various peptides and proteins were found in the nanoparticles (Fig. 2B). A total of 219 proteins were detected, among them, 202 were identified. Subcellular localisation analysis was performed in order to identify the localization of GDNPs proteins within the various subcellular organelle (Fig. 2C). After performing a gene ontology (GO) analysis (Fig. 2D), we confirmed that GDNPs are linked to various biological phenomena, among which several metabolic and cellular processes, possess molecular function such as catalytic activities, and are also related to different types of cellular components. The results of kyoto encyclopedia of genes and genomes (KEGG) analysis (Fig. 2E) showed that GDNPs are critical in signaling pathways related to ribosomes, oxidative phosphorylation, and terpene skeleton biosynthesis. Importantly, they also relate to proteins closely linked to the MAPK signaling pathway. In Additional file 2: Figure S2 and Additional file 3: Figure S3 the further characterization of the proteomics data elucidates the nature of GDNPs proteins. The structure domain prediction software interproscan was used to predict the structure domains of all identified proteins. While the protein interaction network showed in Additional file 4: Figure S4 and  Additional file5 : Table S1 demonstrate close and extensive interactions between the proteins identified.

### In vivo and in vitro uptake of GDNPs by macrophages and GDNPs distribution in vivo

**Fig. 3:**
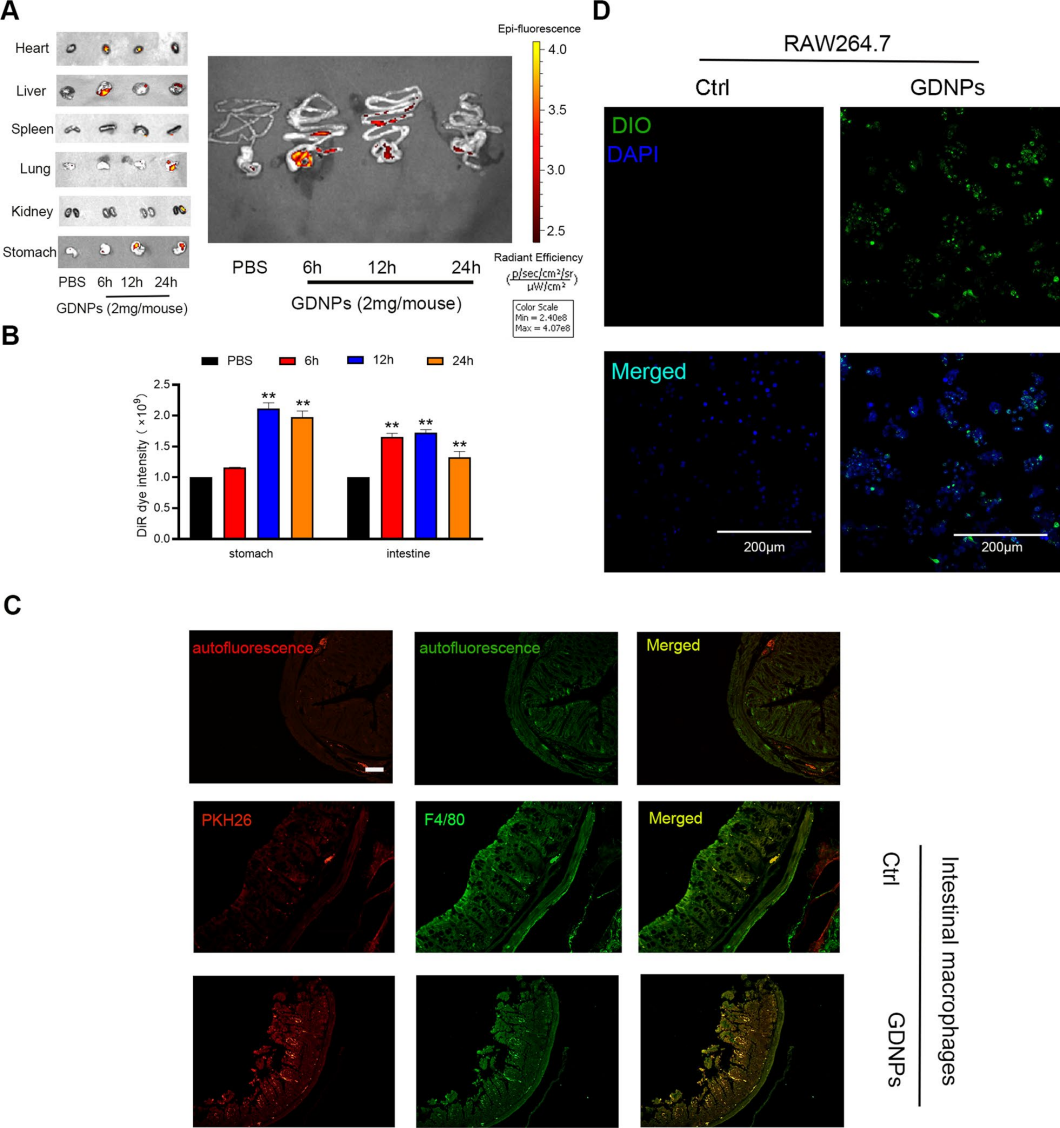
In vitro macrophage uptake. Small animal imaging was used to detect drug distribution in vivo (**A**). PKH-26 dye intensity was measured in stomach and intestine using the Living Image software (**B**). Colocalisation of mouse intestinal macrophages and fluorescently labelled GDNPs. Scale bar: 100 μm (**C**). Confocal images showing uptake of DIO-labeled GDNPs by RAW264.7 in vitro cultures. Scale bar: 200 μm (**D**). Data are expressed as mean ± SD. n = 3. **p < 0.01 vs. PBS group (One-way ANOVA and Dunnett’s post-hoc test)

The in vivo distribution images (Fig. 3A, B) show GDNPs absorption in the heart, liver, spleen, lung, kidney, stomach and intestine after 6 h, 12 h, and 24 h from the administration of PKH-26-labelled GDNPs. GDNPs absorption in the gastrointestinal tract was evident at all timepoints, while the other organs absorbed less pronounced amounts of GDNPs. Most likely, while GDNPs nanoparticles are efficiently absorbed in the gastrointestinal tract, their components moves into circulation and then to other organs after digestion.

Macrophages are present in large numbers in the intestine, and have a vital role in intestinal function and homeostasis. Because of their high phagocytic capacity, intestinal macrophages are very likely to take up GDNPs. Therefore, we performed immunofluorescent colocalisation of PKH-26-labelled GDNPs and intestinal macrophages in mice colorectum (Fig. 3C). We additionally performed an in vitro uptake assay using the macrophage line RAW264.7, We determined that both macrophages in mice colorectum tissue and RAW264.7 cells showed efficient uptake of GDNPs (Fig. 3D).

### GDNPs alleviate LPS-induced mitochondrial dysfunction by reducing oxidative stress levels

**Fig. 4:**
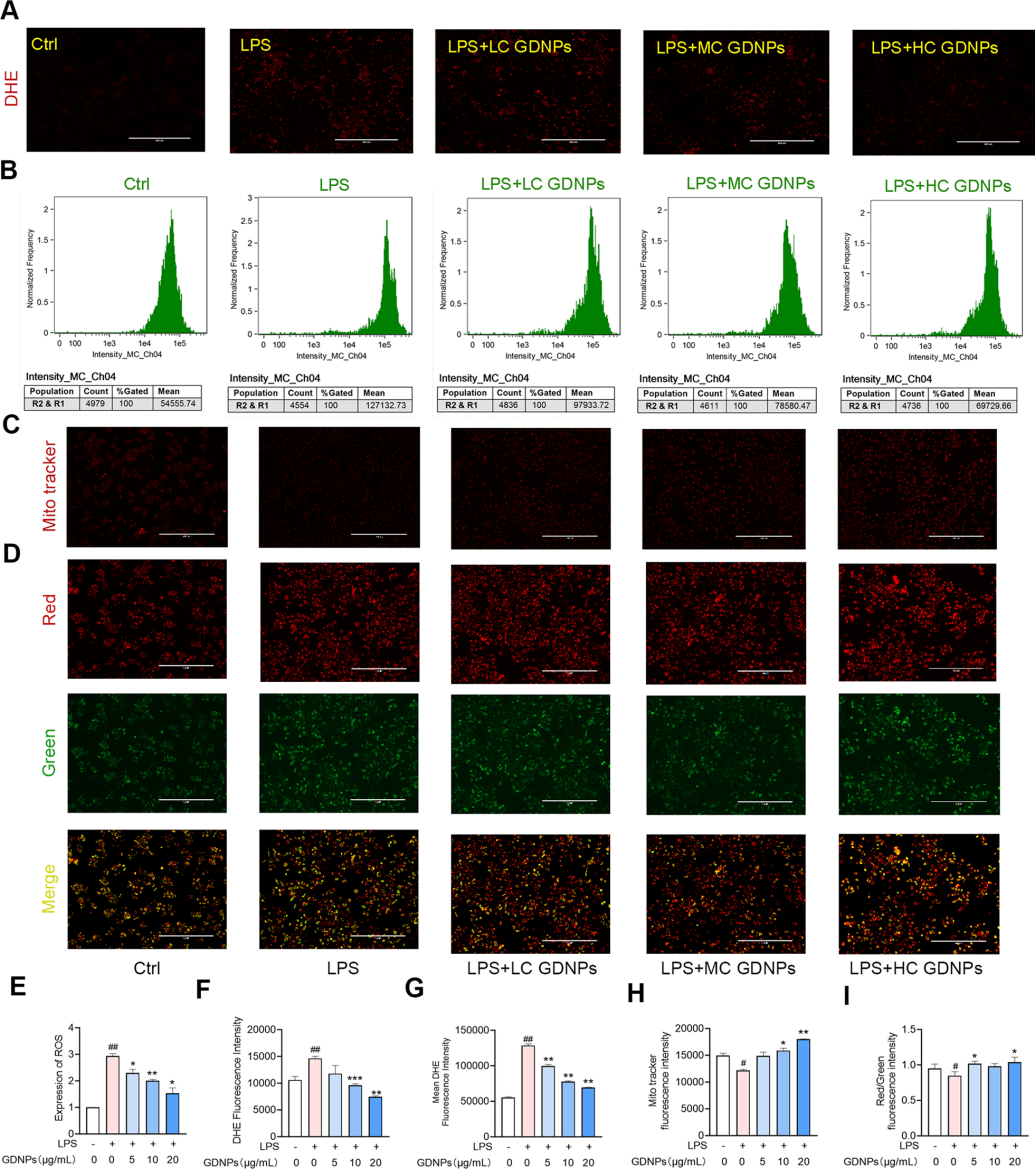
Effects of GDNPs on RAW264.7. Distribution of intracellular DHE under a fluorescence microscope. Scale bar: 400 μm (**A**). Flow cytometry to detect the fluorescence intensity of DHE (**B**). Intracellular mito tracker assay (**C**) and changes in intracellular MMP (**D**) visualized using the red/green ratio. Scale bar: 400 μm. Expression of ROS in the presence of LPS and LPS/GDNPs (**E**). Quantification diagram of DHE by fluorescence microscope (**F**). Quantification diagram of DHE by flow cytometer (**G**). Quantification of mito tracker fluorescence (**H**). Quantification of JC-1 fluorescence (**I**). Image processing was performed using ImageJ analysis software. Data are expressed as mean ± SD. n = 3. ^#^p < 0.05 and ^##^p < 0.01 vs. Control; *p < 0.05 and **p < 0.01 vs. LPS-stimulated cells (One-way ANOVA and Dunnett’s post-hoc test)

Mitochondria are critical organelles for oxygen metabolism. The imbalance of ROS in cells leads to a surge in oxidative stress markers, accelerating the onset and progression of diseases [36].The uptake of GDNPs by macrophages and their colocalisation prompted us to further investigate the effect of GDNPs on macrophages function. We measured the intracellular levels of DHE in RAW264.7 cells treated with GDNPs by fluorescent microscopy (Fig. 4A); and also by flow cytometry (Fig. 4B). These results showed that GDNPs attenuated the elevation of intracellular DHE caused by LPS (Fig. 4F, G). ROS levels (Fig. 4E) were significantly elevated in the inflammation model. GDNPs treatment significantly reduced intracellular ROS formation in LPS-stimulated RAW264.7 macrophages, which is essential for containing oxidative stress.

To determine whether GDNPs can improve mitochondrial function, we investigated mitochondrial number and mitochondrial membrane potential (MMP) in RAW264.7 cells (Fig. 4C, D). Treatment with LPS reduced mitochondrial number, while GDNPs treatment rescued this reduction (Fig. 4H). Alterations of MMP were detected using JC-1. JC-1 is present in the mitochondria of normal cells as a polymer with bright red fluorescence and very weak green fluorescence. Upon decrease in MMP, JC-1 cannot exist in the mitochondrial matrix as a polymer, causing a decrease in the red fluorescence in the mitochondria, while the green fluorescence increases in intensity. As shown in Fig. 4I, GDNPs treatment reverts the decrease in MMP caused by LPS.

### GDNPs activate the p62–Nrf2–Keap1 pathway to attenuate LPS-induced oxidative damage

**Fig. 5:**
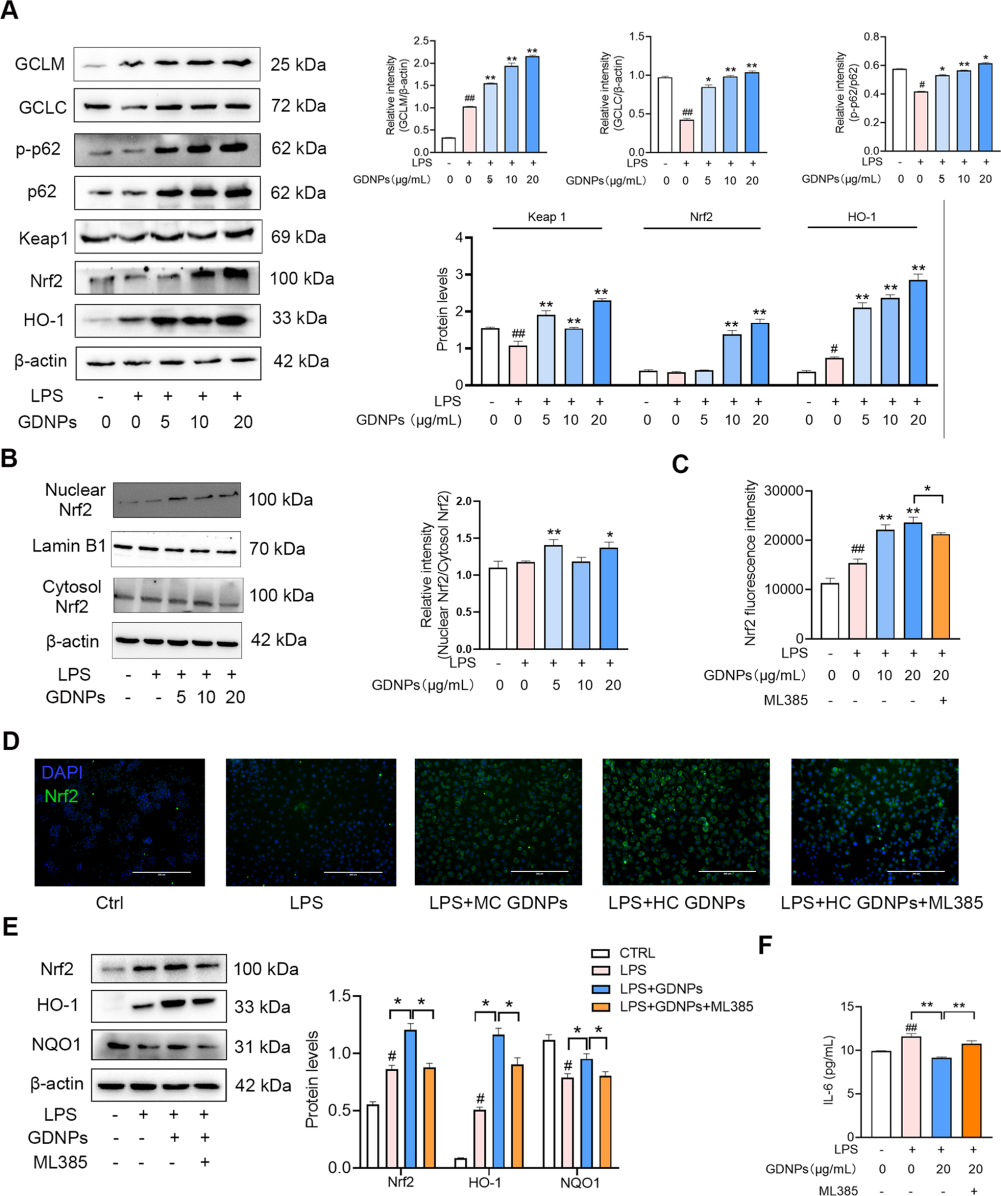
The regulatory effect of GDNPs on the Keap1–Nrf2–p62 signaling pathway in an in vitro model. Protein expression of GCLC, GCLM, HO-1, Nrf2, Keap1, p62, Phospho-sequestosome 1 (p-p62) and β-actin measured by western blotting in RAW264.7 cells treated with and without different concentrations of GDNPs (**A**). Western blot bands demonstrating Nrf2 translocation from cytoplasm to nucleus. **B** Quantitative plot of Nrf2 fluorescence expression (**C**). Nrf2 fluorescence expression in RAW264.7 cells. Scale bar: 400 μm (**D**). Protein expression of HO-1, Nrf2, NQO1 and β-actin measured by western blotting under the action of the inhibitor ML385 in in RAW264.7 cells. **E** Levels of IL-6 in cell supernatants in the presence or absence of the ML385 inhibitor (**F**). Image processing and densitometric analysis were performed using ImageJ analysis software. Data are expressed as mean ± SD. n = 3. ^#^p < 0.05 and ^##^p < 0.01 vs. Control; *p < 0.05 and **p < 0.01 vs. LPS-stimulated cells (One-way ANOVA and Dunnett’s post-hoc test)

Disease development is closely related to inflammation and oxidative stress, and Nrf2 is recognised as an antioxidant factor [37]. To elucidate the antioxidant mechanism of GDNPs, we examined the expression of the p62–Nrf2–Keap1 pathway proteins by western blotting in our LPS-induced inflammation model of RAW264.7 cells. GDNPs significantly promoted the expression of Nrf2 and the levels of its downstream antioxidant enzymes Oxygenase 1 (HO-1), Glutamate-cysteine ligase modifier subunit (GCLC) and Glutamate-cysteine ligase modifier subunit (GCLM) proteins after 24 h of treatment in RAW264.7 cells (Fig. 5A). In addition, GDNPs also increased the protein expression of Sequestosome 1 (p62). High levels of p62 promote the accumulation of Nrf2 [38]. Next, we explored changes in Nrf2 nuclear translocation in response to GDNPs (Fig. 5B). Our results indicated that GDNPs increased Nrf2 localization in the nucleus in order to exert its antioxidant capacity.

To further investigate the mechanisms by which GDNPs reduce oxidative stress and ameliorate IBD, we determined the effects of GDNPs under the interference of the Nrf2 inhibitor ML385 in RAW264.7 cells. Intracellular Nrf2 expression was detected by immunofluorescence (Fig. 5C, D). Our results showed that low and medium concentrations of GDNPs significantly enhanced the level of Nrf2, while this effect was counteracted by ML385. We found that the protein levels of Nrf2 and its downstream factors HO-1 and NAD(P)H quinone dehydrogenase 1 (NQO1) were significantly reduced in the presence of ML385, demonstrating that ML385 significantly inhibited the antioxidant activity of GDNPs (Fig. 5E). Finally, to test whether GDNPs can affect the level of inflammation by modulation of oxidative stress, we used an ELISA kit to measure the level of IL-6 in the supernatant of RAW264.7 cells in the presence of ML385 (Fig. 5F). These data suggest that GDNPs can exert their antioxidant capacity in a in vitro model of IBD by activating the Keap1–Nrf2–p62 pathway.

### GDNPs suppress LPS-induced inflammatory responses by decreasing pro-inflammatory cytokine expression and inhibiting the Toll-like receptor 4 (TLR4)/MAPK pathway

**Fig. 6:**
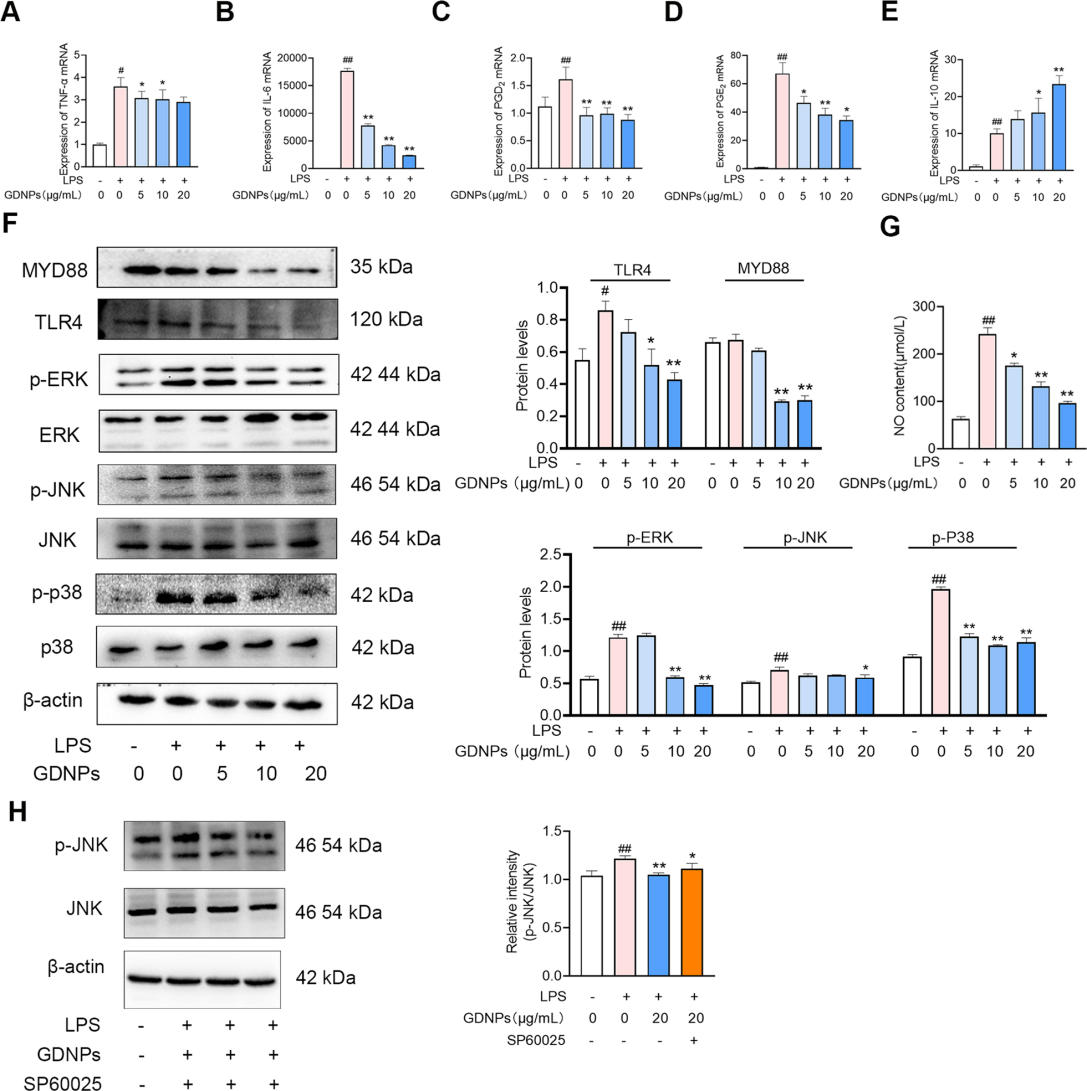
Anti-inflammatory effect of GDNPs in RAW264.7 cells. Changes in intracellular TNF-α (**A**), IL-6, (**B**). PGD_2_ (**C**), PGE_2_ (**D**) and Interleukin 10 (IL-10) (**E**) mRNA levels in LPS-stimulated RAW264.7 cells treated with and without different doses of GDNPs. The protein levels of Myeloid differentiation primary response gene (88) (MYD88), TLR4, ERK, Phospho-extracellular signal-related kinase (p-ERK), JNK, Phospho-c-Jun N-terminal kinase (p-JNK), p38, p-p38 and β-actin were measured by western blotting in LPS-stimulated RAW264.7 cells after treatment with GDNPs (**F**). Expression of NO in the presence of LPS and LPS/GDNPs (**G**). The protein levels of JNK, p-JNK and β-actin were measured by western blotting and compared with the effects of the SP600125 inhibitor (**H**). Densitometric analysis was performed with ImageJ Software. Data are expressed as mean ± SD. n = 3. ^#^p < 0.05 and ^##^p < 0.01 vs. Control; *p < 0.05 and **p < 0.01 vs. LPS-stimulated cells (One-way ANOVA and Dunnett’s post-hoc test)

To further confirm whether GDNPs reduce inflammatory markers, we evaluated TNF-α, IL-6, IL-l0, prostaglandin E2 (PGE_2)_, and prostaglandin D2 (PGD_2)_ in RAW264.7 cells (Fig. 6A–E). As expected, GDNPs significantly inhibited the mRNA levels of pro-inflammatory factors while promoting the expression of anti-inflammatory molecules. Moreover, GDNPs reduced the intracellular levels of NO in our in vitro model (Fig. 6G). Overall, these results suggests that GDNPs play an anti-inflammatory role in LPS-stimulated RAW264.7 cells.

The MAPK pathway is a classical inflammation-related pathway [39]. Western blot results confirmed that the phosphorylation levels of the Extracellular signal-related kinase (ERK), c-Jun N-terminal kinase (JNK), and p38 proteins involved in the MAPK pathway were significantly increased after LPS stimulation of RAW264.7 cells (Fig. 6F). GDNPs reduced the LPS-induced elevated levels of these proteins in a concentration-dependent manner. Similarly, the inhibitor SP600125 reduced the LPS-induced phosphorylation of JNK (Fig. 6H). These data indicate that GDNPs can alleviate LPS-induced inflammation by regulating MAPK signalling.

In the proteomics analysis of GDNPs, our KEGG analysis revealed that GDNPs mechanism of action is linked to the MAPK pathway and oxidative phosphorylation pathway, which validates our in vitro experiment from a different perspective. Together, these results demonstrated that GDNPs treatment diminished inflammation onset and development.

### Protective mechanism of GDNPs on the intestinal barrier in inflammatory states

The first line of intestinal defense against pathogens invasion consists of a layer made of intestinal epithelial cells (IECs), which play an essential role in maintaining the integrity and dynamic balance of the mucosal barrier [40]. When epithelial cells are excessively apoptotic or intestinal tight junctions are impaired, intestinal microorganisms enter the mucosal layer through intestinal leakage, leading to sustained antigenic stimulation, massive recruitment of immune cells, and excessive release of inflammatory mediators [41].

**Fig. 7:**
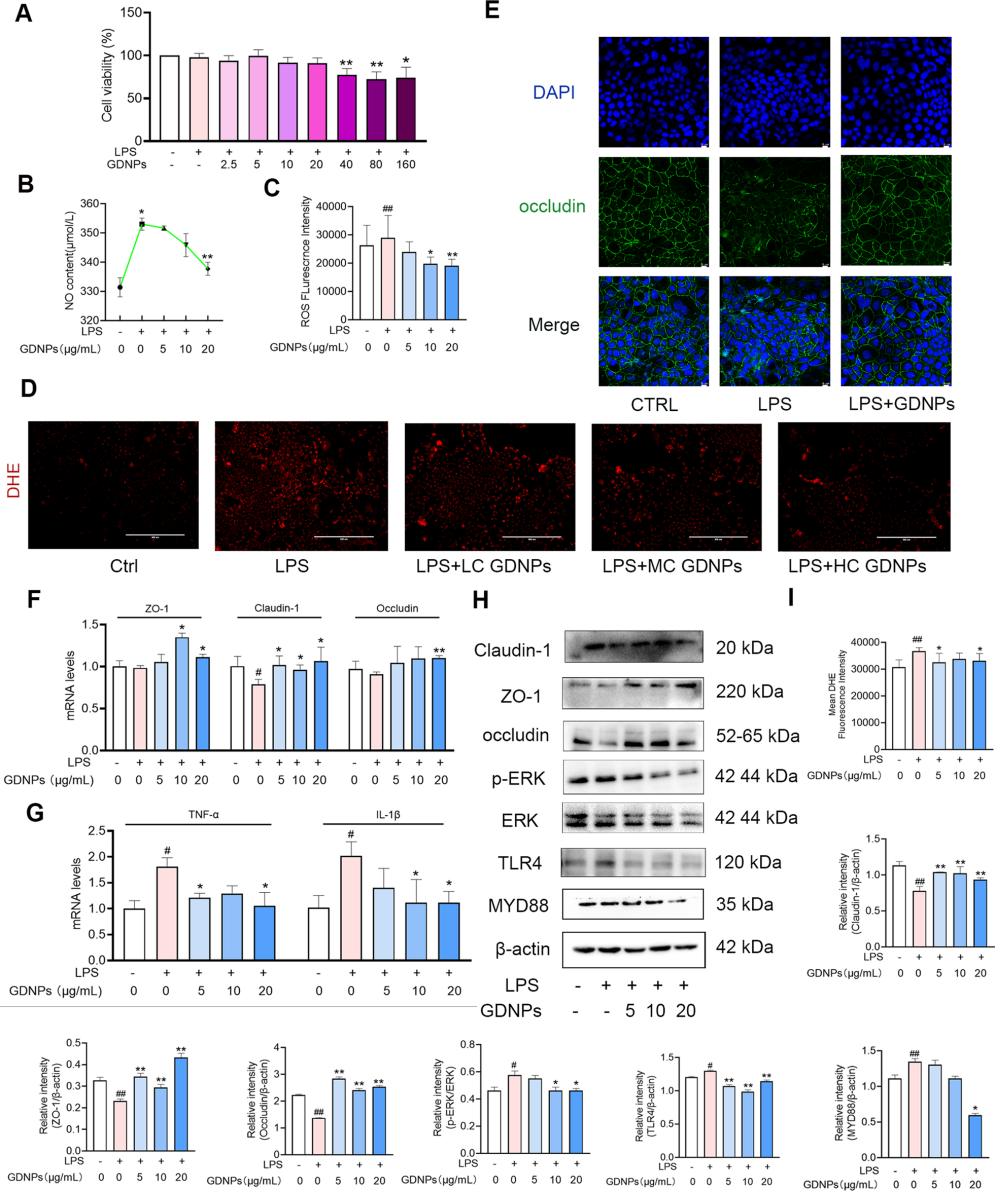
GDNPs repair the first line of defense in the intestinal barrier—intestinal epithelial cells. cck8 assay for the detection of cytotoxicity in Caco-2 cells treated with different concentrations of GDNPs (**A**). Intracellular NO levels (**B**), Intracellular ROS levels (**C**) and DHE fluorescence levels in LPS-stimulated Caco-2 cells with and without different concentrations of GDNPs. Scale bar: 400 μm. **D** Detection of fluorescence intensity of tight junction proteins by immunofluorescence (occludin). Scale bar: 10 μm. **E** Expression of tight junction proteins (ZO-1, occludin, claudin-1) at the transcriptional level (**F**). Expression of inflammation-related proteins at the transcriptional level (**G**). Changes in levels of tight junction proteins and inflammatory proteins (**H**). Quantification of DHE fluorescence intensity (**I**). Image processing and densitometric analysis were performed using ImageJ analysis software. Data are expressed as mean ± SD. n = 3. ^#^p < 0.05 and ^##^ p < 0.01 vs. Control; *p < 0.05 and **p < 0.01 vs. LPS-stimulated cells (One-way ANOVA and Dunnett’s post-hoc test)

Up to this point, we have demonstrated that GDNPs reduce inflammatory factors in immune cells in an in vitro inflammation model. To determine whether GDNPs can repair the intestinal barrier by protecting intestinal epithelial cells, we examined changes in inflammatory factors and tight junction proteins in Caco-2 cells. As shown in Fig. 7, cytotoxicity (Fig. 7A), and levels of NO (Fig. 7B), ROS (Fig. 7C) and DHE (Fig. 7D and I) were first tested. Our results showed that the secretion of NO and the levels of ROS and DHE, gradually decreased upon increasing concentrations of GDNPs. Occludin expression, detected by immunofluorescence, was severely impaired in the tight junctions of LPS-stimulated cells. This impairment was reversed by the action of GDNPs (Fig. 7E). In addition, GDNPs increased the transcript levels of several tight junction proteins [Zonula occludens protein 1 (ZO-1), occludin, claudin-1] (Fig. 7F) and decreased the transcript levels of inflammatory factors (TNF-α, IL-1β) (Fig. 7G). Consistent with these results, tight juctions were upregulated also at the protein levels, while the levels of proteins implicated in the MAPK signaling pathway were significantly diminished (Fig. 7H). Therefore, GDNPs alleviate inflammatory responses in intestinal epithelial cells and enhance the expression level of tight junction proteins by inhibiting ERK in the MAPK /TLR4/MYD88 signaling pathway. Once again in line with our KEGG proteomics results, the MAPK signaling pathway may be an essential target pathway for the function of GDNPs.

### GDNPs enhance intestinal stem cell proliferation and differentiation by activating the Wnt/β-catenin signaling pathway

**Fig. 8:**
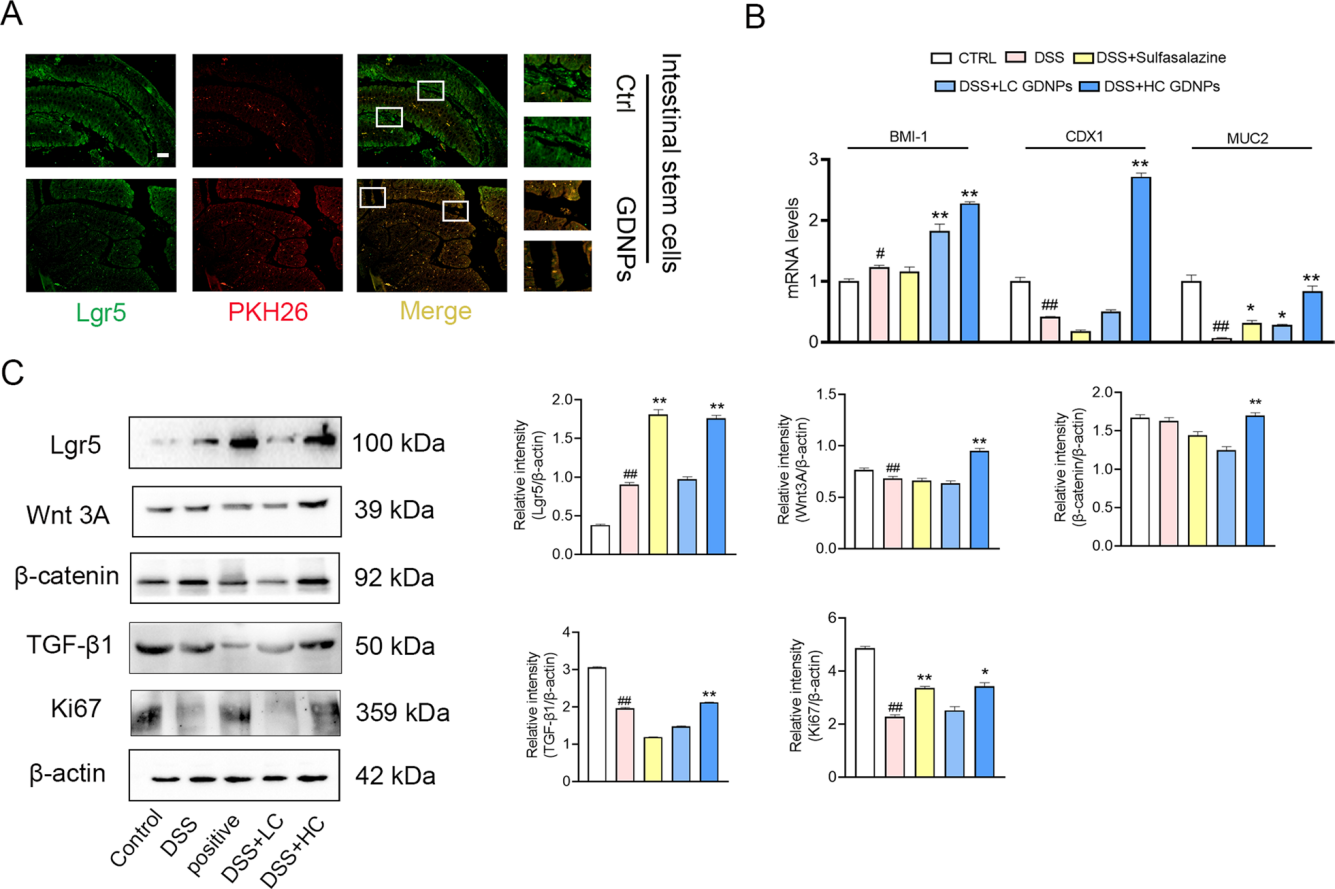
GDNPs regulate intestinal stem cell proliferation and differentiation. Labelled GDNPs colocalise with intestinal stem cells in the mouse intestine. Scale bar: 100 µm. **A** Transcription levels of proliferative and differentiation factors (BMI-1, CDX1 and MUC2) in intestinal stem cells in mice treated with and without GDNPs (**B**). Expression levels of the intestinal stem cell marker Lgr5 and of the proliferative and differentiation protein Wnt 3A, β-catenin, TGF-β1, and Ki67 (**C**). Densitometric analysis was performed with ImageJ Software. Data are presented as mean ± SD. n = 3. ^#^p < 0.05 and ^##^p < 0.01 vs. Control; *p < 0.05 and **p < 0.01 vs. mice treated with DSS only (One-way ANOVA and Dunnett’s post-hoc test)

Homeostasis and continuous tissue replenishment of the intestinal epithelium are driven by the constant division of stem cells located at the bottom of the crypts. The architecture of the crypt-villus axis and the rapid cell turnover allows the intestine to act as both a barrier and a significant site of nutrient uptake [42]. Many signaling pathways regulate cellular self-renewal, including the Wnt/β-catenin, BMP, and Hedgehog signaling pathways [43]. We therefore, examined the stem cells in mouse intestinal crypts and observed colocalisation of labelled GDNPs and intestinal stem cells (Fig. 8A). This colocalisation prompted us to investigate the transcription levels of the stem cell factors involved in proliferation and differentiation, and interestingly, the mRNA levels of BMI-1, caudal type homeobox 1 (CDX1) and mucoprotein 2 (MUC2) were significantly elevated in GDNPs-treated mice, compared to mice treated with only DSS (Fig. 8B). The expression level of Eucine-rich-repeat-containing G-protein-coupled receptor 5 (Lgr5) was significantly higher in the positive control group (DSS + Sulfasalazine) and the high-dose GDNPs group (p < 0.01). Notably, the high-dose GDNPs group had elevated Wnt/β-catenin protein levels and the expression of proliferative and differentiation proteins such as Transforming growth factor beta-1 (TGF-β1) and Ki67 (Fig. 8C). In conclusion, these data suggest that during the repair of the intestinal barrier, GDNPs activate the Wnt/β-catenin signaling pathway in order to promote stem cell proliferation and differentiation, enhancing the renewal capacity of intestinal epithelial cells.

### GDNPs alleviate DSS-induced IBD by activating Nrf2 and inhibiting the MAPK signaling pathway to reduce inflammation and oxidative stress

**Fig. 9:**
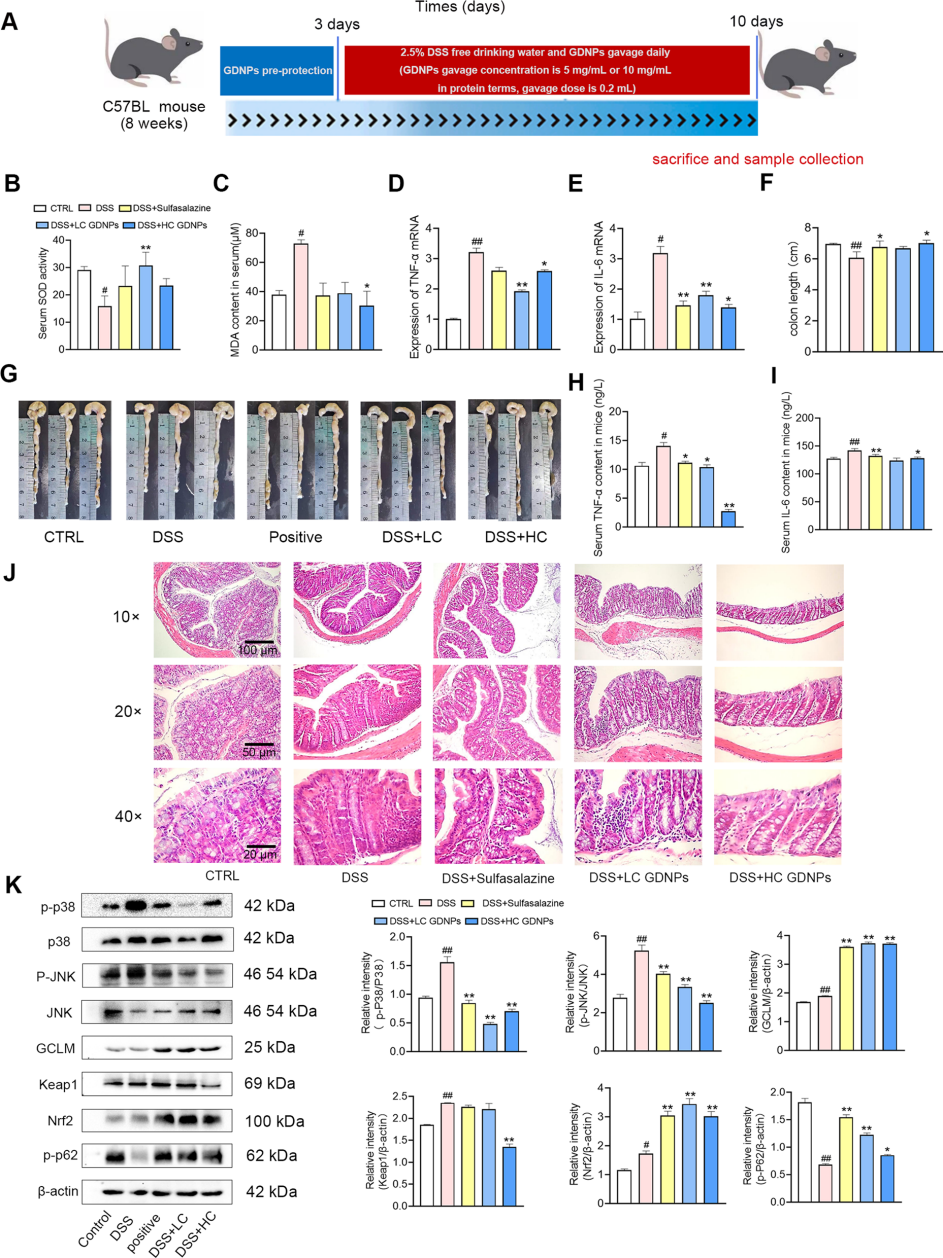
Effect of GDNPs on inflammatory bowel disease in a mouse model. DSS and GDNPs administration regimens in our IBD mouse model (**A**). SOD activity in serum (**B**) and serum MDA levels (**C**) in IBD mice treated with and without GDNPs. TNF-α (**D**) and IL-6 (**E**) mRNA levels in mouse intestinal tissue. Colon length for the different mouse groups (**F**). Representative photographs of intestines for each mouse group (**G**). Serum TNF-ɑ (**H**) and IL-6 (**I**) detected by ELISA. HE staining of Intestine for each mouse group (**J**). Protein expression of p-38, p-p38, JNK, p-JNK, GCLM, Nrf2, Keap1, p-p62 and β-actin in intestinal tissue, measured by western blotting (**K**). Densitometric analysis was performed with ImageJ Software. Data are presented as mean ± SD. n = 3. ^#^p < 0.05 and ^##^p < 0.01 vs. Control; *p < 0.05 and **p < 0.01 vs. mice treated with DSS only (One-way ANOVA and Dunnett’s post-hoc test)

The above findings support an ameliorating role for GDNPs in terms of mediation of inflammatory responses and oxidative stress. To investigate the potential underlying mechanisms of this, we investigated the effect of GDNPs in our DSS-induced IBD mouse model (Fig. 9A). SOD activity, and levels of MDA, TNF-α and IL-6 were measured in serum (Fig. 9B, C, H, I, while TNF-α and IL-6 mRNA levels were measured in intestinal tissues (Fig. 9D, E). These results showed that GDNPs decreased inflammatory cytokine expression, while increasing the antioxidant capacity in vivo. The length of the intestines in each group was measured (Fig. 9F, G) and the intestinal morphology was observed using hematoxylin-eosin staining (H&E) staining (Fig. 9J). Mice in the DSS model group exhibited significant intestinal injury characterised by reduced numbers of epithelial goblet cells and increased infiltration of inflammatory cells between intestinal glands. On the other hand, treatment with GDNPs at high concentrations significantly attenuated intestinal injury in a way similar to sulfapyridine. To determine the specific mechanism by which GDNPs alleviate DSS-induced IBD, we used western blotting to detect the expression levels of proteins involved in the MAPK pathways, and of p-p62, Nrf2, Keap1, and GCLM (Fig. 9K). GDNPs significantly reduced the protein levels of p-P38 and p-JNK, thus these in vivo results recapitulate our previous in vitro results. Overall, these data suggest that GDNPs can exert anti-inflammatory and antioxidant functions by targeting the MAPK and Keap1–Nrf2 pathways in a mouse model of DSS-induced IBD.

### Maintenance of microbiome homeostasis by GDNPs

**Fig. 10:**
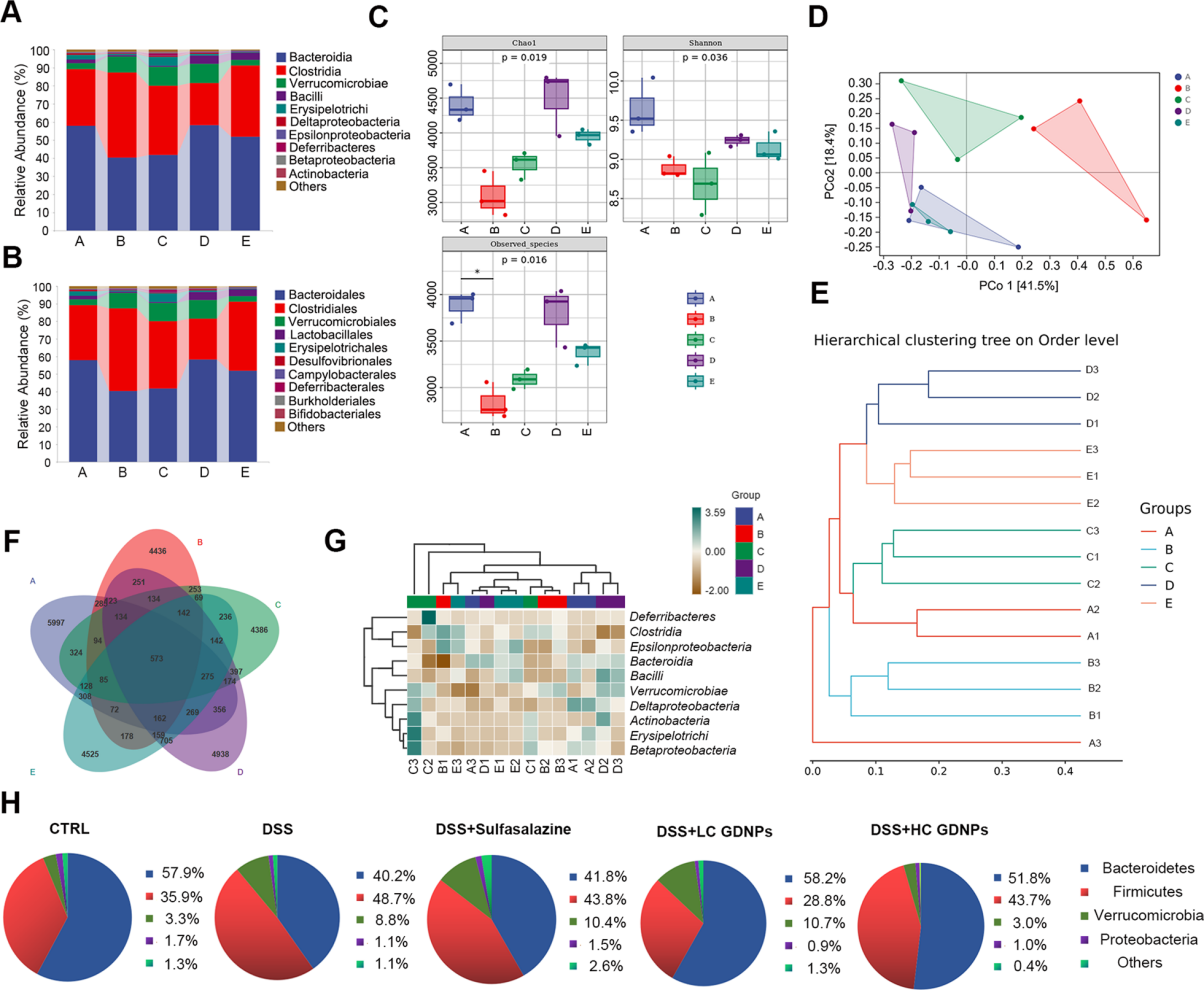
Evaluation of the remodelling effects of GDNPs on the intestinal flora. Abundance of microorganisms in each group at the class level (**A**) and order level (**B**). Chao index, Shannon index and number of observed species of intestinal flora in the different animal groups (**C**). Principal coordinates analysis (PCoA) (**D**). Hierarchical clustering tree on order level (**E**). Venn diagram showing bacterial composition of the microbiome of the different animal groups (**F**). Heat map of species composition at the class level (**G**). The microbial composition of each group at the phylum level (**H**). **A** CTRL group, **B** DSS group, **C** DSS + Sulfasalazine group, **D** DSS + LC GDNPs group, **E** DSS + HC GDNPs group. Data are expressed as mean ± SD. n = 3.*p < 0.05 vs. mice treated with DSS only (Kruskal–Wallis test and Dunnett’s post-hoc test)

IBD is strongly associated with the intestinal flora [44]. Therefore, we investigated whether GDNPs could regulate the homeostasis of mouse intestinal flora. Figure 10A and B show the relative abundance of the different bacterial species in the microbiome of mice treated with and without GDNPs. GDNPs treatment significantly increased species diversity and richness (Chao index, Shannon index and number of observed species) compared to the DSS group (Fig. 10C). The Beta Diversity Index focuses on the comparisons between diverse habitats. Principal co-ordinates Analysis (PCoA) showed that the composition of the intestinal flora differed between the groups. The DSS group was predominantly concentrated in quadrant 2.3. In contrast, the microbiota of the high-dose GDNPs treatment group overlapped with that of the blank control group (Fig. 10D). Cluster analysis reflected a high degree of similarity between the positive control group, GDNPs-treated groups and the blank control group (Fig. 10E). Venn diagram analysis of species composition showed that GDNPs treatment increased the abundance of the gut microbiota (Fig. 10F). The Species composition heatmap shows differences in species composition between groups (Fig. 10G). GDNPs treatment groups had a similar phylum profile to that of the blank control group, further validating the therapeutic efficacy of GDNPs against IBD (Fig. 10H).

The Firmicutes/Bacteroidetes ratio increases during colonic inflammation, and a decrease of this ratio is indicative of the therapeutic efficacy against IBD [45]. Figure 10H shows that the DSS group had a higher Firmicutes/Bacteroidetes ratio compared to the blank control group. In contrast, the GDNPs treatment groups show lower Firmicutes/Bacteroidetes ratios. Short chain fatty acids (SCFAs) are key molecules in the interplay between microbial and host metabolism. Supplements containing *Bacillus sphaericus* protect the integrity of the intestinal epithelium, modulate the microbiota, and increase SCFAs distribution, reducing colonic inflammation [45–47]. In our study, Bacilli were severely absent in the DSS control, but treatment with GDNPs significantly restored their abundance (Additional file 6: Figure S5). The above findings demonstrate the homeostatic regulation of intestinal flora by GDNPs. A stable intestinal flora structure can maintain the strong antimicrobial effect of the intestinal tract, further protecting the intestinal tract from damage.

### In vivo toxicity of GDNPs

**Fig. 11:**
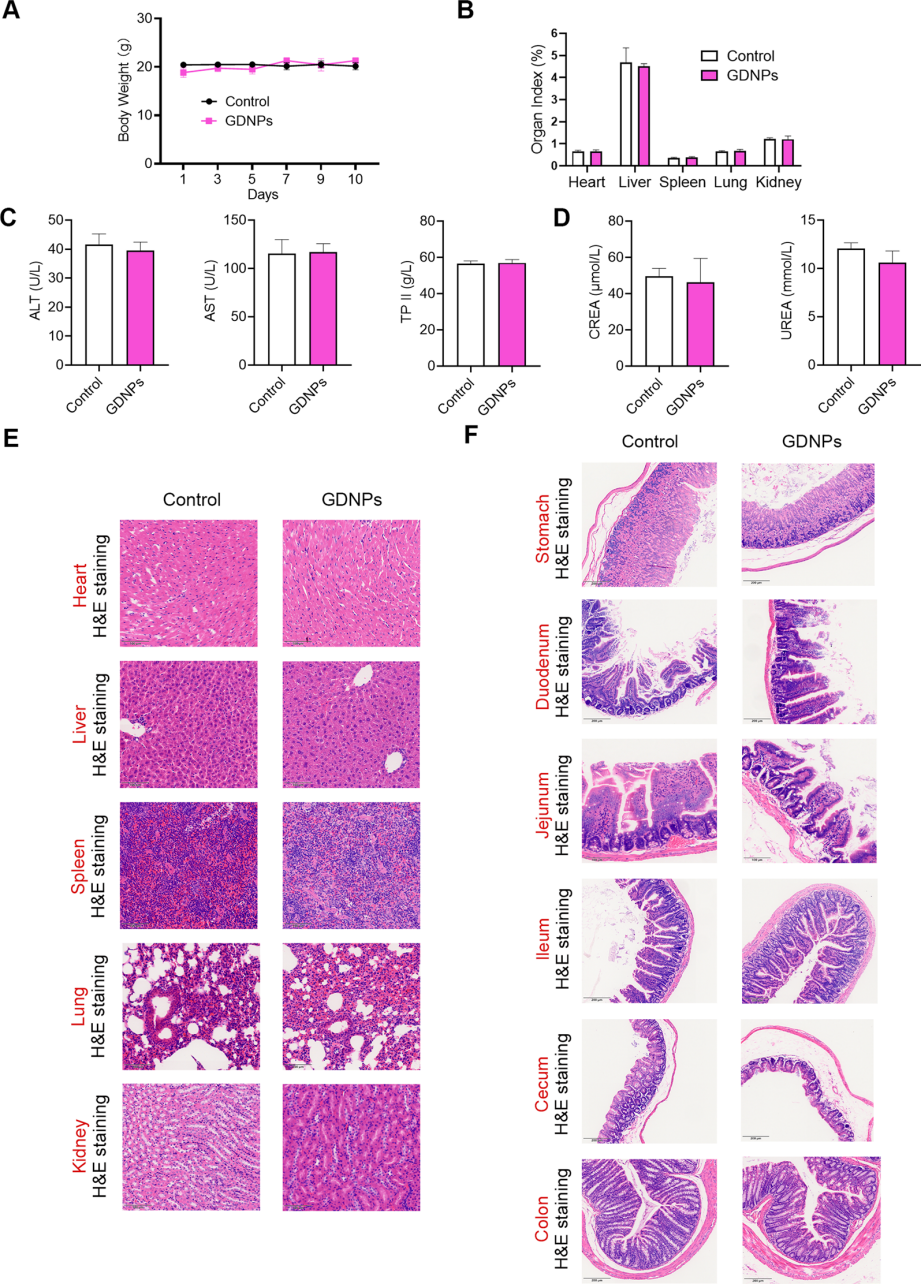
Toxicity assays for GDNPs. Body weight variations in mice treated with and without GDNPs (**A**) and organ (**B**) and serological indices of liver (**C**) and kidney function (**D**). Representative images of H&E-stained liver, kidney, spleen, heart, and lung sections from control and GDNPs-treated mice. Scale bar: 100 µm (**E**). H&E staining of different sections of mouse intestine (stomach, duodenum, jejunum, ileum, cecum, and colon). Scale bar: 200 µm (**F**). Data are presented as mean ± SD. n = 3. No significant difference was shown by Student’s t-test

To determine the systemic toxicity of orally delivered GDNPs in vivo, GDNPs were administered orally by gavage for 10 consecutive days to male C57BL/6J mice. All animals appeared healthy during the whole length of the treatment with GDNPs and showed no significant difference in weight (Fig. 11A). The differences in organ indices were not statistically significant between the two groups (Fig. 11B). GDNPs did not alter liver function-related parameters [Alanine transaminase (ALT), Aspartate transaminase (AST), Total protein (TPII)] and renal function-related indices [Creatinine (CREA), Carbamide (UREA)] compared to controls mice (Fig. 11C, D). To further investigate the hepatorenal toxicity of GDNPs in vivo, mice were sacrificed after 10 days of oral administration and different tissues were collected. H&E stainings (Fig. 11E, F) and blood analysis showed that GDNPs did not cause liver, kidney, spleen, heart or lung damage, and no abnormalities were detected in any of the intestinal sections. These results suggest that oral administration of GDNPs is safe.

## Discussion

In this study, fresh ginseng was used for the extraction of GDNPs. Lipids and proteins are the main bioactive substances in exosomes. Studies have shown that exosomes mediate intercellular communication by transferring active substances to receptor cells to modulate cell function. One example is phosphatidic acid, which naturally promotes the exosome binding to specific receptor cells [48,49] for drug delivery and targeted distribution. Therefore, we hypothesise that the active components in GDNPs may also interact and mediate communication with intestinal cells. Ginseng is mostly used in dried form as medicine and is rich in saponins, polysaccharides, amino acids, etc. However, dried ginseng differs from fresh ginseng, and thus from GDNPs, in terms of active ingredients. This study provides new knowledge in support of fresh ginseng as therapeutic agent.

IBD has become a global disease due to its increasing prevalence in all countries [2]. Commonly used medications to treat IBD include glucocorticoids, antibiotics, and biologics. It is important to note that current treatments cannot cure IBD, which requires lifelong care to prevent or delay the progression of the disease [45]. Therefore, the search for suitable alternative treatments is urgent. Exosomes have been shown to have biological functions such as high biocompatibility and low immunogenicity, and are rich in lipids, proteins, miRNAs and other bioactive components. Negatively charged plant-derived exosomes can easily penetrate the mucus layer to reach the inflammatory regions of the intestine through electrostatic interactions. In addition, plant-derived exosomes can also be loaded with therapeutic chemicals and nucleic acid drugs to deliver their content in a targeted manner that will act stably and efficiently at the site of intestinal inflammation [50, 51]. Because of this particular feature, plant exosomes present important advantages over the more traditional ways of consuming medicinal herbs. Recent studies have shown that the active ingredients in ginseng have a positive effect on chronic inflammation, such as anti-inflammatory and antioxidant effects, which may help to prevent disease progression. Therefore, we investigated the impact of GDNPs in LPS-stimulated RAW264.7 macrophages and intestinal epithelial cells as well as in a mouse model of IBD.

In this study, we examined the size, morphology, lipids and proteins content of GDNPs. We demonstrated that GDNPs can be absorbed by the cells in the intestine, where they attenuate intestinal inflammatory responses, modulate intestinal barriers, enhance stem cell proliferation and differentiation, and maintain microbiome diversity after oral administration. Therefore, it is necessary to explore the mechanism by which GDNPs play a protective role in IBD.

The pathogenic mechanisms of IBD are multifaceted and are related to inflammation, oxidative stress, and imbalance of intestinal flora. Studies have shown that polyphenolic compounds are effective in alleviating intestinal inflammation during the pathogenesis of IBD by modulating antioxidant signaling pathways, such as Nrf2, in order to strengthen the intestinal mucosal barrier, which also favours the homeostasis of the intestinal flora [52]. Nrf2 is a critical transcription factor that induces the expression of antioxidant enzymes and plays a vital role in the antioxidant response [53, 54]. As detected by western blot, GDNPs stimulates the expression of Nrf2 and its downstream effectors Ho-1 and GCLM. p62 interacts with various proteins and is also an activator of the Nrf2 pathway. p62 protects the cells from oxidative stress by sequestering Keap1 and thus freeing Nrf2 from the Nrf2–Keap1 complex. High expression of p-p62 allows Keap1 to be degraded through the autophagic pathway, and leads to the upregulation of Nrf2 downstream antioxidant genes. Therefore, high levels of p-p62 facilitates the scavenging of peroxides in the organism [55]. In our experimental results, p-p62 protein levels were significantly increased in vitro and in vivo. Interestingly, we did not observe a remarkable decrease in Keap1 in our in vitro experiments. We therefore hypothesise that, in this context, GDNPs might have not only induced the autophagic lysosomal degradation of the Keap1 protein, but also inhibited the proteasomal degradation of the Keap1/Nrf2 complex while promoting p62 phosphorylation. Therefore, the observed levels of Keap1 might results from GDNPs acting on both types of protein degradation pathways. However, Keap1 expression decreased in our in vivo animal model, possibly due to the more intense autophagic lysosomal degradation induced in the in vivo experiments. Nevertheless, GDNPs induced strong activation of Nrf2 also in vitro, regardless of the higher observed Keap1 levels. These results suggest that GDNPs exert antioxidant activity by activating the Nrf2 signaling pathway, which contribute to maintain long-term intracellular redox homeostasis. The above results prompted us to further explore the anti-inflammatory effects of GDNPs.

High levels of NO can react with superoxide radicals to generate peroxynitrite, a reactive oxidant associated with several detrimental inflammatory processes. Therefore, lowering intracellular NO levels can inhibit peroxynitrite synthesis and limit the inflammatory processes associated with it [56]. Indeed, studies have shown that down-regulation of nitric oxide synthase expression could improve the conditions of a mouse model of IBD with shortened colon [57]. In our model of inflammation induced by LPS in RAW264.7 cells, we found that NO is elevated in response to LPS, and GDNPs treatment is able to reduce this elevation.

Our qPCR results showed that GDNPs decreased the transcript levels of pro-inflammatory factors while elevating the levels of anti-inflammatory molecules. We could therefore conclude that GDNPs were able to protect RAW264.7 cells from LPS-induced cellular damage by acting on the inflammation-related TLR4/Myd88/MAPK signaling pathway. To verify whether our in vitro conclusions could be translated to an in vivo model, we designed further in vivo experiments.

We used a mouse model of IBD to verify the protective effect of GDNPs. The antioxidant system in the body is divided into enzymatic and nonenzymatic systems. The enzymatic system includes antioxidant enzymes such as SOD and MDA. Our results showed that GDNPs decreased MDA levels and increased SOD activity in mouse serum. Our data confirmed that GDNPs attenuated intestinal symptoms and pathological changes in DSS-induced IBD in mice. Despite this, the mechanisms by which the GDNPs act are still unclear. We therefore further investigated the protective effect of GDNPs on the intestine in vivo and explored the possible molecular mechanisms by which GDNPs alleviate intestinal inflammation. We measured the expression levels in intestinal tissues of key proteins involved in inflammation-related signaling pathways. Interestingly, GDNPs were found to play an essential role in inhibiting the MAPK signalling pathway in vivo, by, among other things, significantly reducing the levels of p-ERK/ERK. Inhibition of the MAPK pathway is indeed an essential target strategy for treating IBD [58]. Likewise, also in our in vivo model, the Keap1/Nrf2 signaling pathway was promoted in the intestine of GDNPs-treated mice. Overall, we have demonstrated that GDNPs ameliorate inflammation and oxidative stress in both in vitro and in vivo models of IBD by activating the Nrf2, and inhibiting the MAPK signaling pathways.

## Conclusion

This study shows that GDNPs ameliorate inflammation in vivo and in vitro. In the present study, we first found that GDNPs inhibited LPS-induced inflammation in RAW264.7 macrophages and intestinal epithelial cells, enhanced the expression of intestinal epithelial cell tight junction proteins, and alleviated DSS-induced IBD in mice. Moreover, we found that GDNPs further activate the expression of downstream antioxidant enzymes by upregulating Nrf2 and inhibiting the TLR4/MAPK signaling pathway. In vivo, GDNPs also ameliorated gut microbiome dysbiosis by inhibiting some potentially harmful pathogens implicated in DSS-mediated inflammatory bowel disease. In addition, GDNPs treatment increased the abundance of beneficial bacteria such as Bacteroidetes, which potentiate the intestinal immune system. In conclusion, GDNPs improved intestinal immunity and gut microbiota composition. Moreover, GDNPs are able to downregulate the TLR4/MAPK signalling pathway while synergistically increasing Nrf2 antioxidant activity to modulate immune responses and alleviate inflammatory bowel disease.

### Supplementary Information

**Additional file 1: Figure S1.** Lipidomics of GDNPs. Lipid content for each lipid class present in GDNPs (ng/g) (A)(B). Quantitative table showing lipid content for all classes (C). Structural formulae for some of the lipids in GDNPs (D).

**Additional file 2: Figure S2.** Proteomic studies of GDNPs. Flowchart of proteomics experiments (A). Structural domain analysis diagram (B).

**Additional file 3: Figure S3.** Proteomic studies of GDNPs. Relative molecular mass distribution of GDNPs proteins (A). Distribution of the number of identified peptides in GDNPs (B).

**Additional file 4: Figure S4.** Protein interaction network.

**Additional file 5: Table S1. **Nomenclature and functional categorisation of proteins contained in GDNP.

**Additional file 6: Figure S5.** Relative abundance of intestinal flora among the different mouse groups. Data are presented as mean ± SD. n = 3; #p < 0.05 vs. Control, *p < 0.05 vs. mice treated with DSS only (One-way ANOVA and Dunnett’s post-hoc test).

## Data Availability

The datasets generated during and/or analyzed during the current study are available from the corresponding author on reasonable request.

## Abbreviations

ALT: Alanine transaminase
ANOVA: Analysis of variance
AST: Aspartate transaminase
CDX1: Caudal type homeobox 1
CREA: Creatinine
DAPI: 2-(4-Amidinophenyl)-6-indolecarbamidine dihydrochloride
DCFH-DA: 2ʹ,7ʹ-Dichlorodihydrofluorescein diacetate
DIO: 3,3′-Dioctadecyloxacarbocyanine perchlorate
DNA: Deoxyribonucleic acid
DPBS: Dulbecco’s phosphate-buffered saline
DSS: Dextran sulfate sodium salt
ECL: Enhanced chemiluminescence
ERK: Extracellular signal-related kinase
GCLC: Glutamate-cysteine ligase catalytic subunit
GCLM: Glutamate-cysteine ligase modifier subunit
GDNPs: Ginseng-derived nanoparticles
GO: Gene ontology
H&E: Hematoxylin-eosin staining
HC: High concentration
HO-1: Oxygenase 1
HRD1: HMG-CoA reductase degradation 1
IBD: Inflammatory bowel disease
IECs: Intestinal epithelial cells
IL-1β: Interleukin-1β
IL-6: Interleukin 6
IL-l0: Interleukin 10
ITS: Internal transcribed spacer
IVIS: In vivo imaging system
JC-1: A probe of mitochondrial membrane potential
JNK: C-Jun N-terminal kinase
Keap1: Kelch Like ECH Associated Protein 1
KEGG: Kyoto encyclopedia of genes and genomes
LC: Low concentration
LC–MS/MS: Liquid chromatography-tandem mass spectrometry
Lgr5: Eucine-rich-repeat-containing G-protein-coupled receptor 5
LPS: Lipopolysaccharide
MAPK: Mitogen-activated protein kinase
MC: Middle concentration
MDA: Malondialdehyde
MMP: Mitochondrial membrane potential
MTBE: Methyl tert-butyl ether
MUC2: Mucoprotein 2
MyD88: Myeloid differentiation primary response gene (88)
NF-κB: Nuclear factor kappa-B
NO: Nitric oxide
NQO1: NAD(P)H quinone dehydrogenase 1
Nrf2: Nuclear factor erythroid2-related factor 2
p62: Sequestosome 1
PA: Phosphatidic acid
PBS: Phosphate buffer saline
PBST: Phosphate buffered saline with tween-20
PCoA: Principal coordinates analysis
p-ERK: Phospho-extracellular signal-related kinase
PGD_2_: Prostaglandin D2
PGE_2_: Prostaglandin E2
p-JNK: Phospho-c-Jun N-terminal kinase
p-p62: Phospho-sequestosome 1
qRT-PCR: Quantitative real-time polymerase chain reaction
RNA: Ribonucleic acid
ROS: Reactive oxygen species
SCFAs: Short chain fatty acids
SDS-PAGE: Sodium dodecyl sulfate polyacrylamide gel electrophoresis
SOD: Superoxide dismutase
TEM: Transmission electron microscopy
TGF-β1: Transforming growth factor beta-1
TLR4: Toll-like receptor 4
TNF-ɑ: Tumor necrosis factor-α
TPII: Total protein
UREA: Carbamide
ZO-1: Zonula occludens protein 1
