# Supplementary figures and images for: Ginseng-derived nanoparticles alleviate inflammatory bowel disease via the TLR4/MAPK and p62/Nrf2/Keap1 pathways
Source: J Nanobiotechnology. 2024 Feb 1;22:48. doi: 10.1186/s12951-024-02313-x (PMC10832157; doi:10.1186/s12951-024-02313-x)

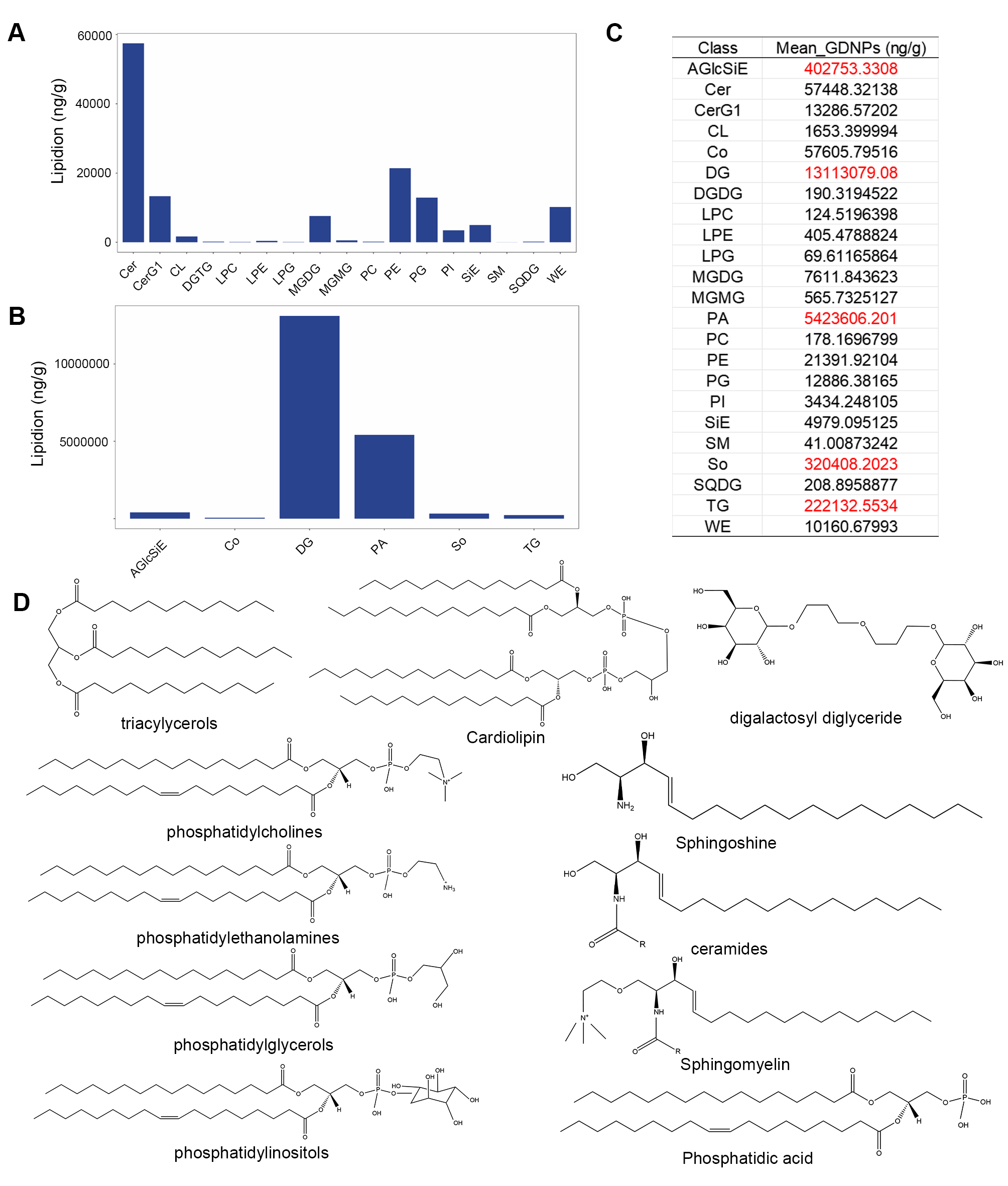

Supplement: Supplementary file 1 — Additional file 1: Figure S1. Lipidomics of GDNPs. Lipid content for each lipid class present in GDNPs (ng/g) (A)(B). Quantitative table showing lipid content for all classes (C). Structural formulae for some of the lipids in GDNPs (D). [file 12951_2024_2313_MOESM1_ESM.tif]

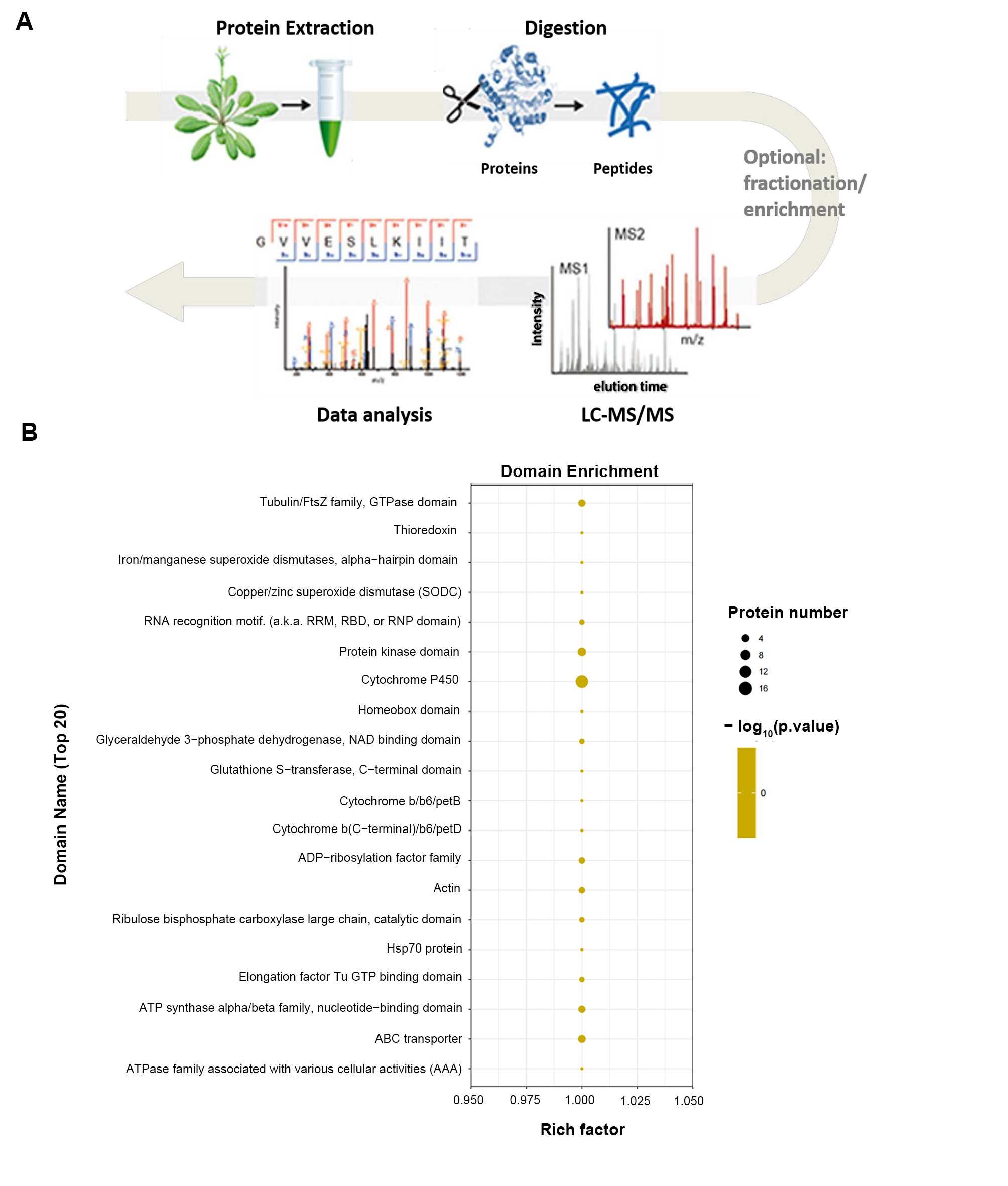

Supplement: Supplementary file 2 — Additional file 2: Figure S2. Proteomic studies of GDNPs. Flowchart of proteomics experiments (A). Structural domain analysis diagram (B). [file 12951_2024_2313_MOESM2_ESM.tif]

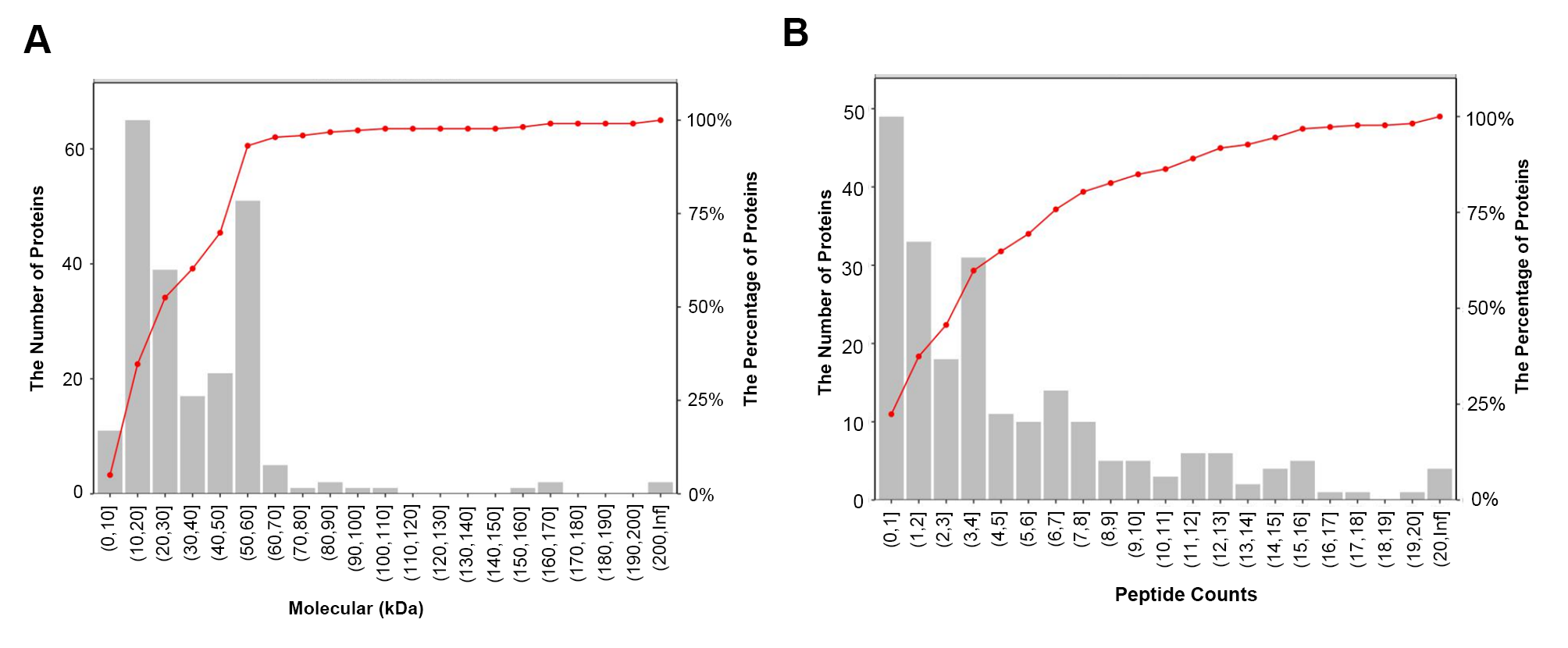

Supplement: Supplementary file 3 — Additional file 3: Figure S3. Proteomic studies of GDNPs. Relative molecular mass distribution of GDNPs proteins (A). Distribution of the number of identified peptides in GDNPs (B). [file 12951_2024_2313_MOESM3_ESM.tif]

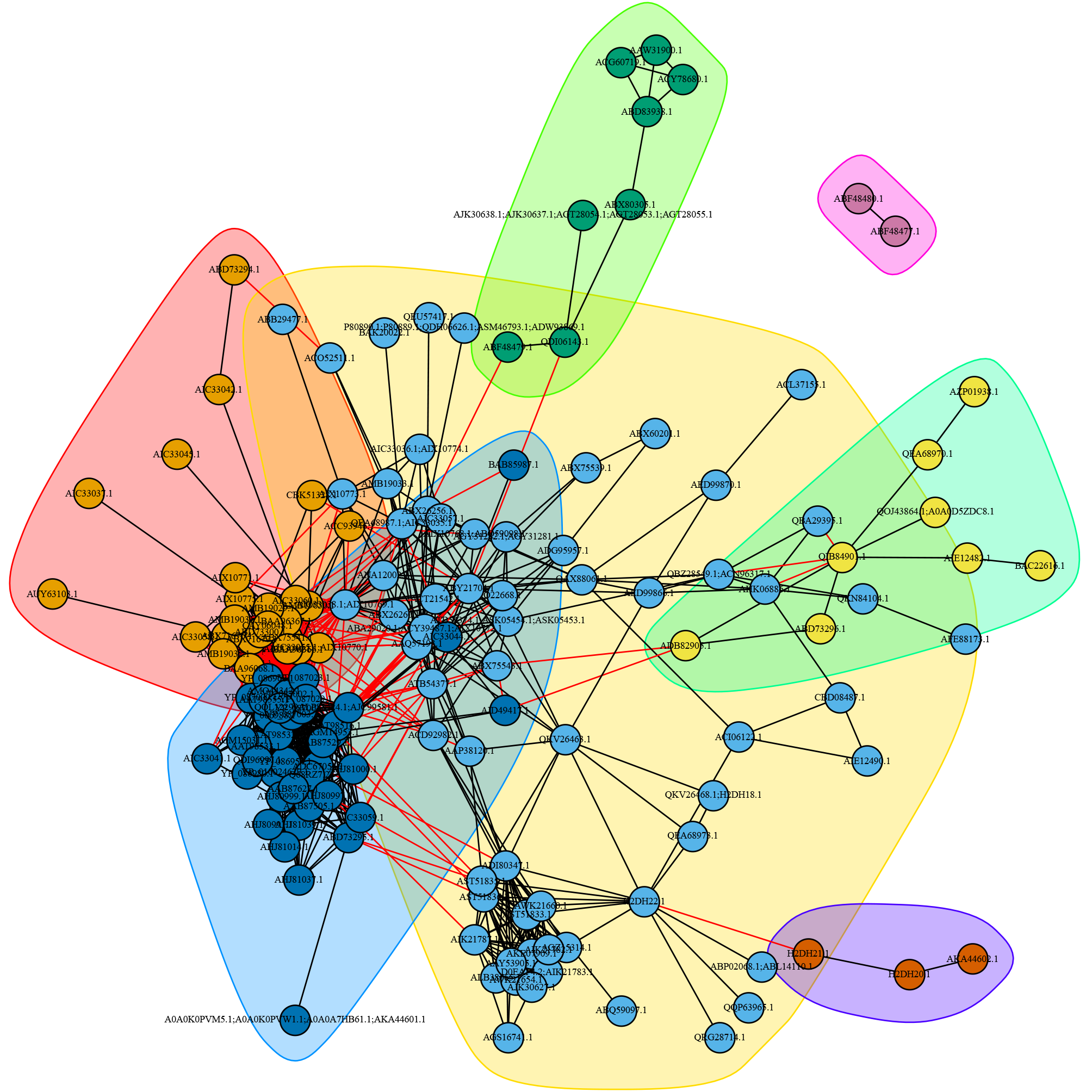

Supplement: Supplementary file 4 — Additional file 4: Figure S4. Protein interaction network. [file 12951_2024_2313_MOESM4_ESM.tif]

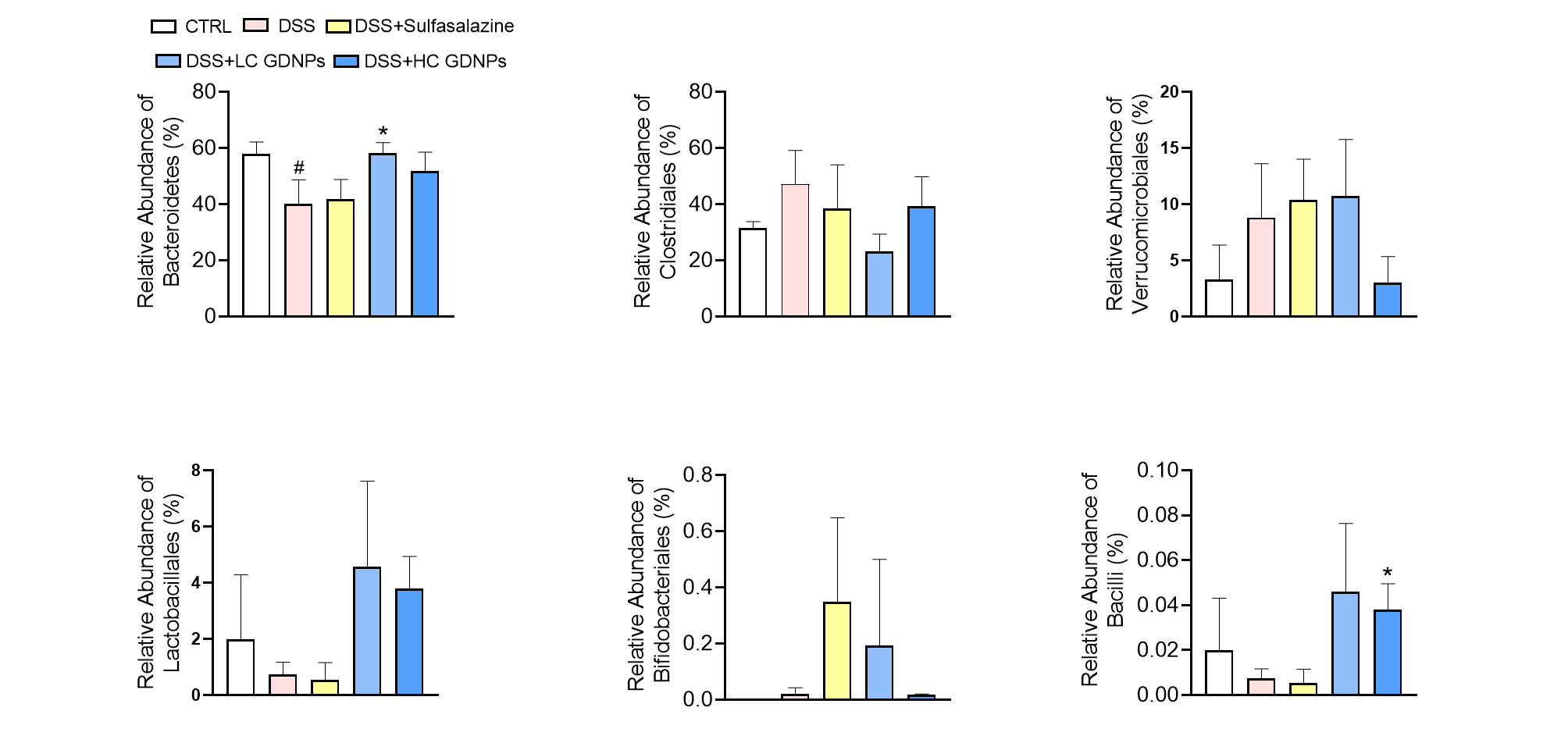

Supplement: Supplementary file 6 — Additional file 6: Figure S5. Relative abundance of intestinal flora among the different mouse groups. Data are presented as mean ± SD. n = 3; #p < 0.05 vs. Control, *p < 0.05 vs. mice treated with DSS only (One-way ANOVA and Dunnett’s post-hoc test). [file 12951_2024_2313_MOESM6_ESM.tif]
